# Screening for potential nuclear substrates for the plant cell death suppressor kinase Adi3 using peptide microarrays

**DOI:** 10.1371/journal.pone.0234011

**Published:** 2020-06-02

**Authors:** In-Cheol Yeo, Timothy P. Devarenne

**Affiliations:** Department of Biochemistry & Biophysics, Texas A&M University, College Station, Texas, United States of America; Universita degli Studi di Padova, ITALY

## Abstract

The tomato AGC protein kinase Adi3 is a Ser/Thr kinase that functions as a negative regulator of programmed cell death through cell death suppression (CDS) activity in the nucleus. In this study, to understand the mechanism of Adi3 CDS, peptide microarrays containing random Ser- and Thr-peptide phosphorylation substrates were used to screen for downstream phosphorylation substrates. In the microarray phosphorylation assay, Adi3 showed promiscuous kinase activity more toward Ser-peptides compared to Thr-peptides, and a preference for aromatic and cyclic amino acids on both Ser- and Thr-peptides was seen. The 63 highest phosphorylated peptide sequences from the Ser-peptide microarray were selected as queries for a BLAST search against the tomato proteome. As a result, 294 candidate nuclear Adi3 substrates were selected and categorized based on their functions. Many of these proteins were classified as DNA/RNA polymerases or regulators involved in transcription and translation events. The list of potential Adi3 substrates was narrowed to eleven and four candidates were tested for phosphorylation by Adi3. Two of these candidates, RNA polymerase II 2^nd^ largest subunit (RPB2) and the pathogen defense related transcription factor Pti5, were confirmed as Adi3 phosphorylation substrates by *in vitro* kinase assays. Using a mutational approach two residues, Thr675 and Thr676, were identified as Adi3 phosphorylation sites on RPB2. This study provides the foundation for understanding Adi3 CDS mechanisms in the nucleus as well as other cellular functions.

## Introduction

Programmed cell death (PCD) is indispensable for appropriate cell growth, development, cell homeostasis, and sculpting of organs or body parts for eukaryotes [1]. PCD events in prokaryotic cells are required for adaptations to stressful environments such as nutrient deprivation through formation of multicellular fruiting bodies and sporulation [2]. In mammalian systems, protein kinase B (PKB, a.k.a. Akt), is a crucial negative regulator of PCD [3, 4]. PKB negatively controls pro-apoptotic factors such as BAD and caspase-9 [5], while activating apoptosis inhibitors such as NF-κB and BCL-2 [6]. Moreover, PKB plays a role in host defense against bacterial infections. PKB is preferentially expressed in neutrophils, which are early immunological effectors against invading pathogens, and PKB expression is down-regulated in response to bacterial infection to stimulate neutrophil functions [7].

PKB belongs to the AGC family of protein kinases. AGC kinases are highly conserved among eukaryotes and are one of the most well characterized families of protein kinases due to their crucial roles in processes such as cell death, protein synthesis, gene transcription, cell growth and division, and cytoskeletal remodeling [8–11]. AGC kinases share sequence similarity in their catalytic domains with the foundational members of this family: cAMP-dependent protein kinase 1 (PKA), cGMP-dependent protein kinase (PKG), and protein kinase C (PKC) [12], hence the name AGC kinases.

In plants, although PCD is required for proper growth and development, one of the more commonly studied PCD functions is the elimination of damaged and infected cells in response to abiotic and biotic stresses [13–15]. In terms of biotic stresses, pathogens have developed virulence molecules called effectors, which are secreted into the plant cell to suppress the host early immunity responses and PCD [16]. However, plants have developed resistance (R) proteins to sense these pathogen-derived effectors. This perception induces the hypersensitive response (HR) characterized in part by localized host PCD to prevent the successful colonization and spread of pathogens [1, 17].

We have characterized a PKB-like negative regulator of PCD in tomato plants termed AvrPto-dependent Pto-interacting protein 3 (Adi3) that controls PCD during the resistance response of tomato to the bacterial pathogen *Pseudomonas syringae* pv. *tomato* (*Pst*) [18–24]. As with PKB, Adi3 is a Ser/Thr protein kinase belonging to the AGC kinase family, and specifically belongs to the plant specific group VIII subfamily [18].

Adi3 acts as a negative regulator of PCD through its activity of cell death suppression (CDS), and entry into the nucleus is required for its CDS activity [18, 23]. Recently, it was shown that Adi3 traffics from the plasma membrane to the nucleus via retrograde transport through the endomembrane system [23]. However, Adi3 is restricted to the endosomal system in response to biotic stresses such as *Pst* and abiotic stresses such as heat and wounding [23]. This regulation of Adi3 cellular localization prevents Adi3 from entering the nucleus and eventually leads to a loss of Adi3 CDS and induction of PCD such as the HR [23].

As described above, Adi3 has analogous functional properties to mammalian PKB as a negative regulator of PCD [3, 4, 18]. As with all AGC kinases, both Adi3 and PKB are regulated by the upstream kinase 3-phosphoinositide-dependent protein kinase-1 (Pdk1) [18]. Furthermore, both protein kinases negatively regulate PCD through the control of MAPK signaling cascades [18, 25]. Although many pro-apoptotic and anti-apoptotic substrates regulated by PKB have been identified [4], only one Adi3 phosphorylation substrate has been identified [20]. We have found that Galactose Metabolism 83 (Gal83), which is a β-subunit of the SnRK1 complex that regulates carbon metabolism and stress responses [26], is phosphorylated Adi3 [20]. Thus, the downstream signaling pathways for Adi3, especially identification of nuclear substrates, are still not known.

Therefore, to understand Adi3 CDS regulation in the nucleus via phosphorylation events, we have used Ser- or Thr-peptide microarrays to screen for putative nuclear substrates of Adi3. The results show that Adi3 has promiscuous protein kinase activity toward a variety of Ser- and Thr-peptides, and Adi3 may regulate diverse cellular functions beyond PCD through nuclear phosphorylation events.

## Material and methods

### Cloning, expression, and mutagenesis of recombinant proteins

To express Adi3, Gal83, and putative Adi3 substrates, cDNAs were cloned into the pMAL-c2x vector (New England BioLabs) for an N-terminal maltose binding-protein (MBP) fusion protein as previously described [20]. The constructs were expressed in *E*. *coli* BL21 (DE3) and purified using amylose resin (New England BioLabs) following the manufacturer’s instructions. Point mutants in Adi3, Gal83, and putative Adi3 substrates were generated by site-directed mutagenesis (SDM) using Pfu Turbo DNA polymerase (Stratagene). SDM on domain 3 of RPB2 was performed using non-overlapping primer sets following the protocol from Dominy and Andrews [27]. Once amplification products were generated with the non-overlapping primers, the products were phosphorylated and ligated to form a circular plasmid using T4 Polynucleotide Kinase and T4 DNA ligase, respectively, (New England BioLabs) prior to transformation into *E*. *coli*. All primers used in this study for cloning and SDM are listed S1 Table.

### *In vitro* kinase activity assay

*In vitro* kinase assays were carried out in a total final volume of 30 μL in a kinase buffer containing 10 mM Tris-HCl, pH 7.5, 150 mM NaCl, 10 mM MgCl_2_, and 1 mM DTT. Reactions including 1 μg of Adi3 and 3 μg of each substrate were started with the addition of 1 μCi of [γ-^32^P]ATP (6,000 Ci/mmol, Perkin-Elmer) and non-radiolabeled ATP to a final concentration of 20 μM per reaction followed by incubation for 1 hour at RT. Reactions were terminated by the addition of 10 μL 4X SDS-PAGE sample buffer and separated by 8% SDS-PAGE. The proteins in the gels were visualized using GelCode Blue Stain Reagent (Thermo Fisher Scientific), and gels were dried and exposed overnight to a phosphor screen. Visualization and quantification of incorporated radioactivity were conducted using a phosphorimager (Typhoon FLA7000, GE Healthcare Life Sciences) and quantification software (ImageQuant TL, GE Healthcare Life Sciences).

The Adi3 kinase assays with Gal83 substrate (see S1A Fig) were initially done in our previous study [28]. These assays were repeated here because the kinase assay conditions in the current study have changed since our previous study. In the previous study [28], 0.4 μg of Adi3, 2 μg of Gal83, and 0.25 μCi of [γ-^32^P]ATP were used and reactions were incubated for 30 min. All of these amounts and the reaction time were increased in the current study as described above. While there were differences in Gal83 absolute phosphorylation levels between the two sets of data, the trends are the same: Adi3^S212D/S539D^ showed the highest *trans*-phosphorylation on Gal83 in both experiments.

### Kinase activity assay on the microarray chip

Peptide phosphorylation microarray chips (JPT Peptide Technologies) with Ser and Thr phosphorylation sites were used in this study. Kinase-active Adi3^S212D/S539D^ was used to phosphorylate peptides in these chips. To stimulate kinase activity, 20 μg of Adi3 was pre-incubated in a total volume of 500 μL of kinase buffer containing 10 mM Tris-HCl, pH 7.5, 150 mM NaCl, 10 mM MgCl_2_, 1 mM DTT, 3 μM Na_3_VO_4_, and 20 μM non-radiolabeled ATP for 30 min at RT. In this step, non-radiolabeled ATP was supplied to stimulate and saturate Adi3 autophosphorylation activity. To activate Adi3-mediated *trans-*phosphorylation of peptides on the microarray chip, 50 μCi of [γ-^32^P]ATP (6,000 Ci/mmol, Perkin-Elmer) was added to the previous 500 μL kinase reaction to give a final volume of 505 μL, which was incubated with the microarray chip for 3 hours at RT. The microarray chips were washed 5 times with 0.1 M phosphoric acid to stop the reaction and remove excess unincorporated [γ-^32^P]ATP. Finally, the chips were washed with methanol and completely dried under nitrogen gas. Confirmation of incorporated radioactivity was performed by exposing the microarray chip to a phosphor screen for 24 hours and imaging with a phosphorimager as described above. A total of five Ser-peptide chips and four Thr-peptide chips were phosphorylated for use in this study. Four of these Ser-peptide chips were used to standardize phosphorylation conditions and for the comparison of kinase activity between Adi3^S539D^ and Adi3^S212D/S539D^. One Ser-peptide chip was used for phosphorylation by Adi3^S212D/S539D^ to identify peptide sequences for identification of potential substrates and comparison to Thr-peptide chip phosphorylation. Of the four Thr-peptide chips phosphorylated by Adi3^S212D/S539D^, only the one chip with the highest resolution and the lowest background noise was used to for comparison to Ser-peptide chip phosphorylation.

### Phosphorylated peptide chip image analysis and data evaluation

Analysis of the phosphorimage to identify phosphorylated peptides from the phosphorylated peptide chip microarray was conducted by JPT Peptide Technologies. The microarray image was analyzed using spot-recognition software, GenePix Pro (Molecular Devices), to identify signal intensity (relative units, RU) which revealed similar patterns of activity in the three subarray regions. A grid file including information of peptide location was overlaid on the microarray phosphorimage to identify peptides phosphorylated by Adi3. To distinguish real signals from background noise the mean signal intensities were analyzed by kernel density estimates [29]. An arbitrary threshold was fixed as being two times the standard deviation above the maximum of density distribution. See results section for more details about setting the threshold limit.

### Identification of amino acid preferences in Adi3 phosphorylated peptides

In order to determine amino acid preference in peptides phosphorylated by Adi3, the sequences from the top 63 peptides showing the highest mean signal intensity in the first and second subarray images of the phosphorylated Ser-peptide chip were used. To analyze the amino acid composition of the top 63 peptides, the percentage of a single amino acid within the 63 peptide sequences was compared to the percentage of the respective amino acid in the whole peptide library. Additionally, to determine position-dependent amino acid preference within the top 63 peptides, the frequency of each amino acid at a given position was counted and divided by the total count of the respective amino acid. This was done for the composition of the top 63 peptides and for all peptides present in the library. Finally, the position-dependent value for all peptides was subtracted from the value for the top 63 peptides. To determine amino acid positional frequencies within the phosphorylated peptides, sequence logos of the top 10, 20, 30, 40, 50, or 63 peptides on the Ser-peptide chip and top 10 peptides on the Thr-peptide chip were generated using the WebLogo 3 server [30].

### Bioinformatic analysis

The top 63 peptides phosphorylated by Adi3 were used for subsequent identification of potential substrate candidates. The amino acid sequences of these 63 peptides were used for a BLASTP search against the tomato proteome in the NCBI database to identify potential nuclear substrates for Adi3.

## Results

### Peptide phosphorylation microarray chips

**Fig 1:**
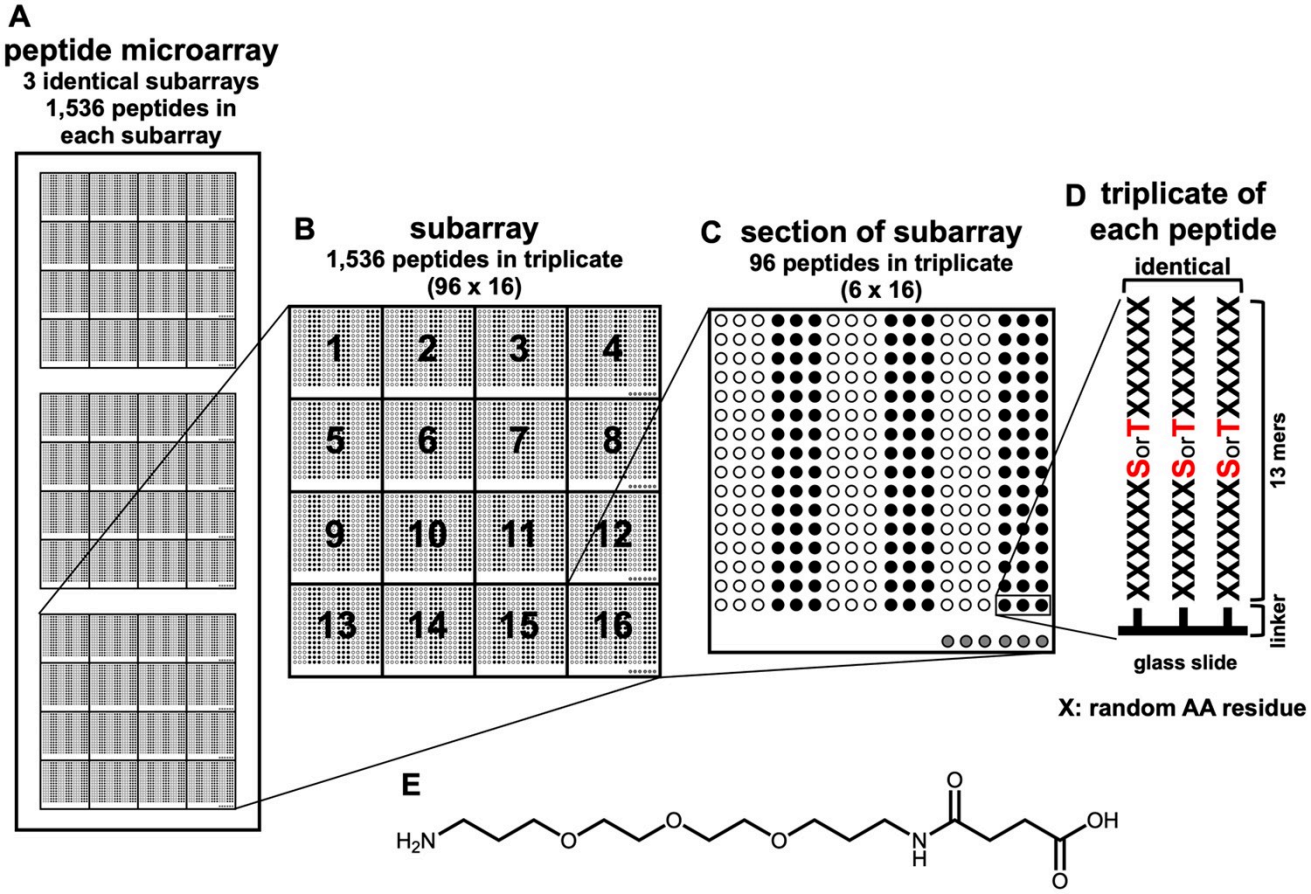
Schematic layout of the peptide microarray chip. (A) The peptide chip consists of three identical subarrays. (B) Each subarray has 1,536 peptides divided among 16 sections. (C) Each peptide is spotted in triplicate, and collectively, each peptide is immobilized on the peptide chip in nine replicates. (D) Each 13 amino acid peptide containing one Ser or Thr residue in the center position peptide is immobilized to the glass slide via a (E) Ttds-linker at the N-terminus of immobilized peptide.

To screen for possible Adi3 nuclear phosphorylation substrates, Ser- and Thr-peptide microarray chips were utilized. Each microarray chip consists of three identical subarray (SA) regions (Fig 1A) and each SA contains 1,536 unique peptides spotted in 16 subsections (Fig 1B). Within each subsection of the SA each peptide is spotted in triplicate (Fig 1C). Thus, each peptide is represented in nine replicates across the whole chip. Each peptide is a random 13-mer peptide containing a central Ser or Thr residue for phosphorylation (Fig 1D). The peptides are immobilized onto the glass surface at the N-terminus (Fig 1D) via a linker of trioxatridecan-succinamic acid (Ttds; Fig 1E). Cys is not present in the peptide library because of its susceptibility towards oxidation.

### Selection of Adi3^S212D/S539D^ as the kinase for peptide microarray phosphorylation

Our previous studies have shown that Adi3 is phosphorylated at Ser539 by Pdk1, the upstream kinase for AGC family kinases [18]. This Pdk1-mediated phosphorylation event is responsible for full CDS activity and nuclear entry of Adi3 [19]. We have also identified an additional Pdk1-mediated phosphorylation site on Adi3, Ser212 [22]. Gal83, a β-subunit of the tomato SnRK1 complex [26], is the only known substrate for Adi3 and is phosphorylated by Adi3 at Ser26 [20]. This second Pdk1 phosphorylation on Adi3, Ser212, in addition to Ser539 is required for its full kinase activity toward Gal83 [22].

To confirm whether the double phosphomimetic mutant Adi3^S212D/S539D^ could act as an effective protein kinase to screen substrates on the peptide microarray, the *in vitro* phosphorylation activity of the phosphomimetic Adi3^S212D/S539D^ on Gal83 was compared to wild-type and two single Adi3 phosphomimetic mutants, Adi3^S212D^ or Adi3^S539D^. The results of these *in vitro* kinase assays show that Adi3^S212D/S539D^ displayed a two-fold increase in phosphorylation of Gal83 over wild-type, and was higher than both Adi3^S212D^ or Adi3^S539D^ (S1A Fig). To determine whether Adi3^S212D/S539D^ also showed higher kinase activity toward peptides on the microarray chip, Adi3^S539D^ and Adi3^S212D/S539D^ were incubated with a Ser-peptide chip in an *in vitro* kinase assay. The results indicate Adi3^S212D/S539D^ was able to phosphorylate the peptides stronger as well as phosphorylate more and different peptides as compared to Adi3^S539D^ (S1B and S1C Fig). Thus, Adi3^S212D/S539D^ was selected as the kinase to phosphorylate the peptide microarray for subsequent use in identifying potential substrates.

### Adi3 shows preference for Ser peptide phosphorylation on the peptide microarray

**Fig 2:**
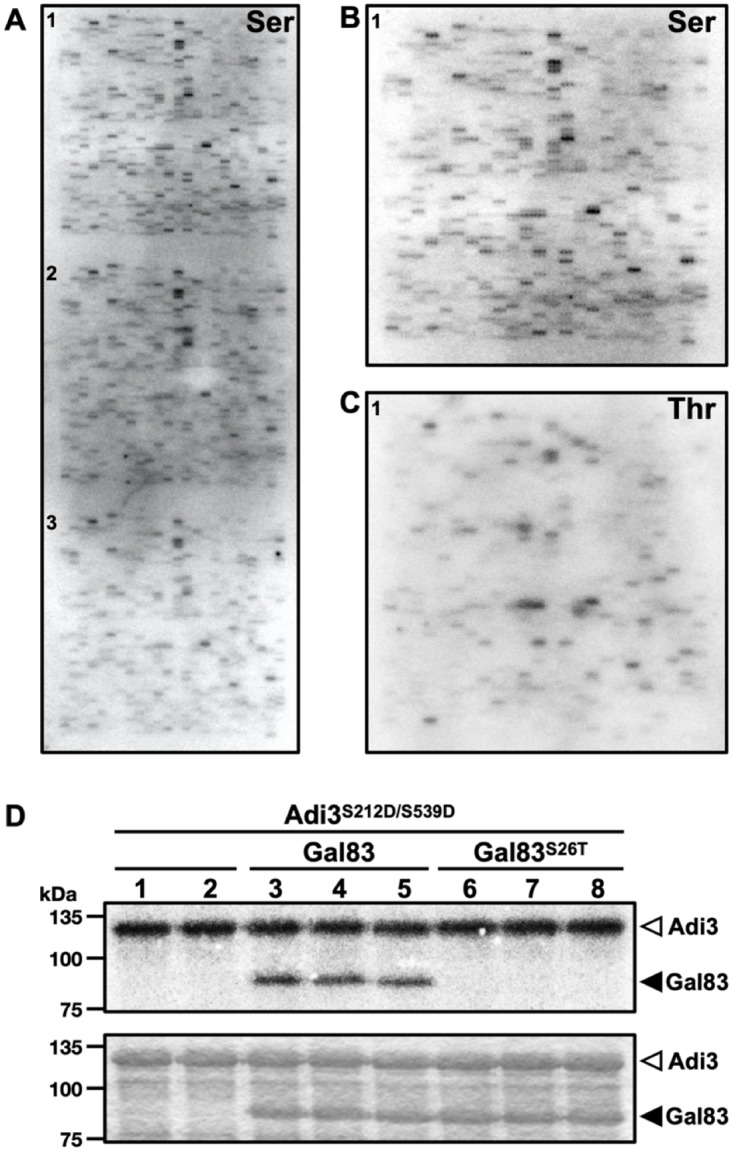
The Adi3 phosphorylated peptide chips and comparison of kinase activity of the Adi3^S212D/S539D^ mutant on Ser and Thr residues. (A) Phosphorimage of the whole Ser-peptide microarray chip. Numbers represent each subarray region. Image shown is representative of 4 Ser-peptide chips phosphorylated by Adi3^S212D/S539D^. (B) and (C) show one subarray region of Ser- and Thr-peptide microarray chips, respectively. (D) *in vitro* Adi3 phosphorylation of Gal83 and Gal83^S26T^ to analyze Adi3 kinase activity on both Ser and Thr residues. Adi3 was incubated with [γ-^32^P]ATP in the absence (lane 1, 2) and presence of Gal83 (lane 3 to 5) or Gal83^S26T^ mutant (lane 6 to 8). Top and bottom panels indicate the phosphorimage and Coomassie stained gel, respectively.

The Adi3^S212D/S539D^ protein was used to phosphorylate both Ser- and Thr-peptide microarray chips to determine if there is a preference of Adi3 for Ser or Thr phosphorylation. For these assays, Adi3^S212D/S539D^ was incubated with the peptide chips and ^32^P-ATP for 3 hours followed by imaging with a phosphorimager. Phosphorylation of the Ser-peptide chip showed consistent phosphorylation across all three subarrays of the chip in terms of intensity and the peptides phosphorylated (Fig 2A). The first subarray of the phosphorylated Ser-peptide chip showed the clearest visualization of each phosphorylated peptide and had the lowest background (Fig 2B). Thus, this subarray was chosen for identification of the phosphorylated peptide sequences and for a comparison to the phosphorylated Thr-peptide microarray. Phosphorylation of the Thr-peptide chip also showed consistent phosphorylation between each subarray of the chip (S1D Fig). One of the phosphorylated subarrays of the Thr-peptide chip is shown in Fig 2C.

Following the analysis and comparison of the phosphorylated Ser- and Thr-peptide microarray chips several interesting results were obtained. For example, more peptides were phosphorylated on the Ser-peptide chip, 345, compared to the Thr-peptide chip, 127 (S2A and S2B Fig), with 107 peptides shared between the two chips, 238 peptides phosphorylated only on the Ser chip and 20 peptides only phosphorylated on the Thr chip (S2C Fig). On the Ser-peptide chip, the peptides were ranked by phosphorylation level from high to low (see below for details). Out of the top 20 of these peptides, 18 were also phosphorylated on the Thr-peptide chip (S3A, S3B and S3C Fig). Out of all the peptides phosphorylated on the Thr-peptide chip, only 10 peptides were not shared with the Ser-peptide chip (S3B and S3D Fig). These results indicate that although Adi3 is a Ser/Thr protein kinase, it shows a higher Ser-specific kinase activity over that of Thr phosphorylation activity. It should be noted that many of the peptides phosphorylated on both chips contain additional Ser and/or Thr residues in addition to the central Ser or Thr target (S3C and S3D Fig). Thus, for these peptides it is difficult to determine which amino acid(s) is being phosphorylated.

The preference of Adi3 for Ser phosphorylation over Thr phosphorylation was supported by analyzing the phosphorylation of the Adi3 substrate Gal83. As indicated above, Adi3 was found to phosphorylate only Ser26 in Gal83 [20]. This residue was mutated to Thr, Gal83^S26T^, and the ability of Adi3 to phosphorylate Gal83^S26T^ in an *in vitro* kinase assay was tested. Interestingly, Adi3 was not able to phosphorylate Gal83^S26T^ (Fig 2D), again supporting a preference for Ser phosphorylation for Adi3. For this reason, the phosphorylated peptides on the Ser-peptide chip were chosen for identifying potential Adi3 substrates.

### Selection of the 63 peptides with the highest Adi3 phosphorylation level

By a naked-eye visual inspection, 345 of the 1,536 peptides (22.5%) on the Ser chip were phosphorylated (S2A Fig). Prior to using the sequence of these phosphorylated peptides to identify potential Adi3 substrates, the phosphorylated peptides were ranked in order of strength of phosphorylation, from high to low. To do this, the signal intensities of all 1,536 peptides in the Ser-peptide chip in subarrays 1 and 2 were measured. Subarray 3 was not included in the analysis since the phosphorylation levels were relatively lower than subarrays 1 and 2 (Fig 2A). Thus, a mean signal intensity value for each peptide was determined from the six replicates of each peptide in the analysis. The minimum mean signal intensity was 2,435 relative units (RU) in the 1,536^th^ peptide and a maximum of 7,853 RU was seen in the first peptide (S4A Fig, S1 Dataset). Most of the mean signals were distributed around 4,000 RU (S4A Fig). To further gain confidence in real signals and distinguish them from background noise, which gives detectable signal to non-phosphorylated peptides, a threshold was set by analyzing signal intensities by kernel density estimates. The standard deviation (SD) of the mean signal intensity values of all 1,536 peptides was calculated and the threshold was determined as two times the SD above the maximum density distribution: 4,000 RU + (2 x SD value). This translates to a threshold mean intensity of 5,384 RU (dotted vertical magenta line in S4A Fig), and signals at or above that threshold can be considered originating from well-distinguishable Adi3 phosphorylation events. Of all the phosphorylated peptides, 63 peptides were above that threshold (S4A Fig). The selected 63 peptides were mapped on the Ser-peptide microarray image (S4B Fig) and their sequences are listed in S2 Table. All 1,536 peptides are listed in S1 Dataset and are ranked by their mean signal intensities. After these first 63 peptides, it can be seen on the phosphorimage that more peptides also showed recognizable signal intensities above the background (S4B Fig). Thus, the next 101 highest phosphorylated peptides (see S1 Dataset) were also selected for possible use in identifying potential Adi3 substrates.

### Analysis of sequence conservation among the top 63 peptides phosphorylated by Adi3

**Fig 3:**
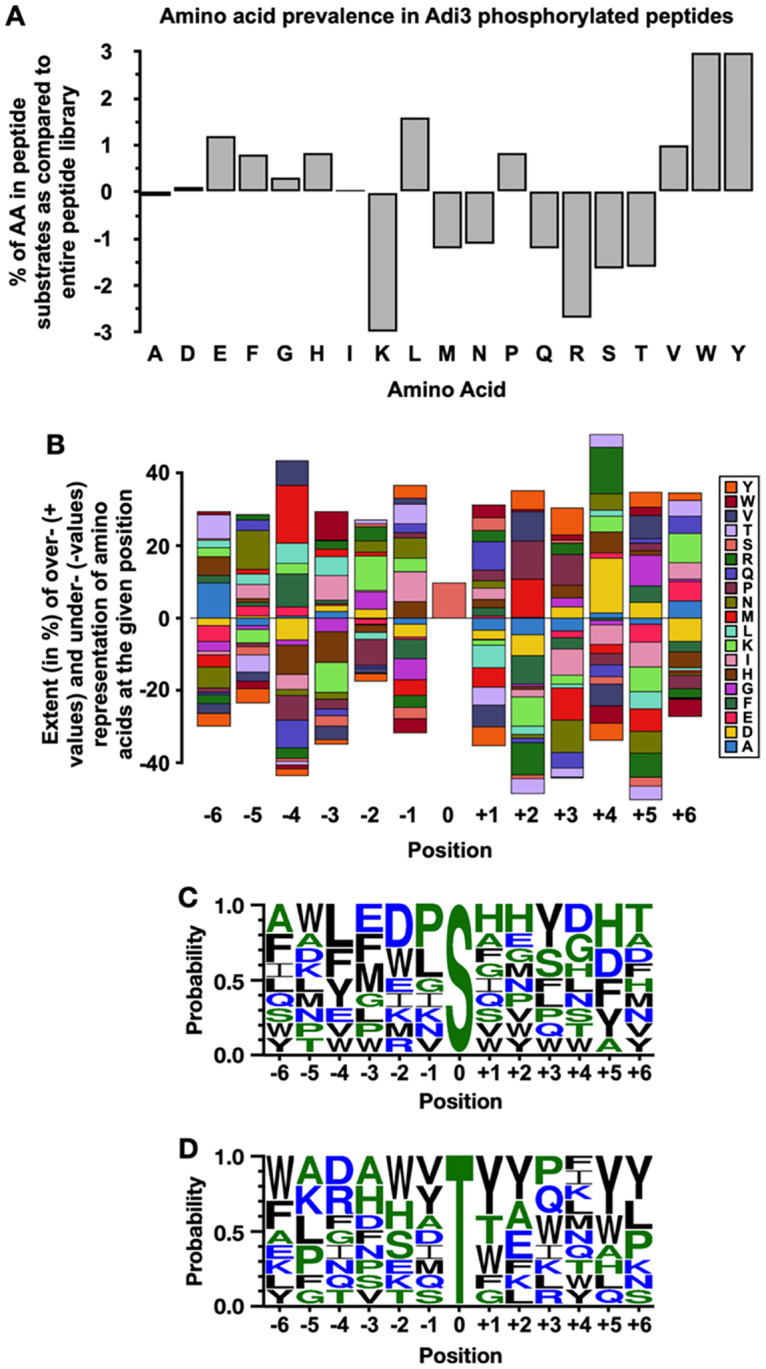
Analysis of amino acid preferences in Adi3 phosphorylated peptides. (A) Distribution of amino acid composition of the top 63 peptides comparted to entire peptide microarray library. Over- and under-representation of amino acids results in a positive value and a negative value, respectively. (B) Stack-plots of position-dependent deviations of frequencies for single amino acids. The height of the bars indicates the extent (in %) of over- or under-represented individual amino acids in the top 63 peptides as compared to the composition of all peptides in the microarray library. In C and D, amino acid positional probability consensus with top 10 phosphorylated peptides from the (C) Ser- and (D) Thr-peptide microarrays using Sequence logo (WebLogo 3). The size of the amino acid code in the sequence logo represents the frequency of that amino acid at a particular position.

In order to identify any possible conserved sequence motifs for Adi3 phosphorylation sites, the sequence of the top 63 phosphorylated peptides was analyzed. First, the percentage of each amino acid within the top 63 phosphorylated peptides was compared to the percentage of each amino acid among all peptides present in the library revealing over- or under-representation for each amino acid in the phosphorylated peptides. The most abundant amino acids are non-polar amino acids containing an aromatic ring, Trp and Tyr (Fig 3A). The positively charged amino acids Lys and Arg are under-represented among the top hits (Fig 3A). Interestingly, cyclic amino acids are over-represented such as Phe, His, Pro, Trp, and Tyr (Fig 3A).

Next, to determine position-dependent amino acid preference, the frequency of each amino acid at a given position within the 63 phosphorylated peptides was counted and that position-relevant value was divided by the total count of the respective amino acid in all peptides in the library to show over- or under-representation at each position. The results show that amino acids with aromatic rings, Trp and Tyr, are over-represented at sites downstream of the central Ser, especially at position +2, +3, and +5 (Fig 3B). Interestingly, Pro, which is an over-represented amino acid in the position-independent analysis (Fig 3A), is also favorably used after the central Ser at positions +1, +2, and +3 (Fig 3B). Since Adi3 phosphorylated many peptides on the microarray chip an obvious consensus sequence for Adi3 phosphorylation was not seen from this analysis. To try and overcome this ambiguity, only the sequences of the top 10 peptides were analyzed using the online sequence logo generator WebLogo [30] to identify a potential Adi3 phosphorylation consensus sequence. From this analysis, a strong consensus sequence is still not obvious (Fig 3C). However, it appears Adi3 may prefer amino acids with cyclic structures (His, Tyr, Trp, Pro) and acidic amino acids (Asp and Glu) at positions both up- and down-stream of the central Ser residue (Fig 3C). This analysis was extended by grouping the Adi3 phosphorylated peptides into the top 20, 30, 40, 50 and 63 peptides. While cyclic and acidic amino acids are still prevalent there is no obvious consensus sequence from this analysis (S5 Fig).

The same analysis was carried out for the top 10 peptides only phosphorylated on the Thr-peptide microarray chip (S3B and S3D Fig). As with the Ser phosphorylation consensus sequence, there is no obvious conserved Thr phosphorylation consensus sequence for Adi3. However, several Tyr residues downstream of the central Thr (+1, +2, +5, and +6 positions) were significantly conserved, and two Trp residues upstream of the central Thr (-2 and -6 positions) appeared to be conserved (Fig 3D).

### Identification of potential Adi3 nuclear substrates by BLAST search using the top 63 phosphorylated peptides as queries

**Fig 4:**
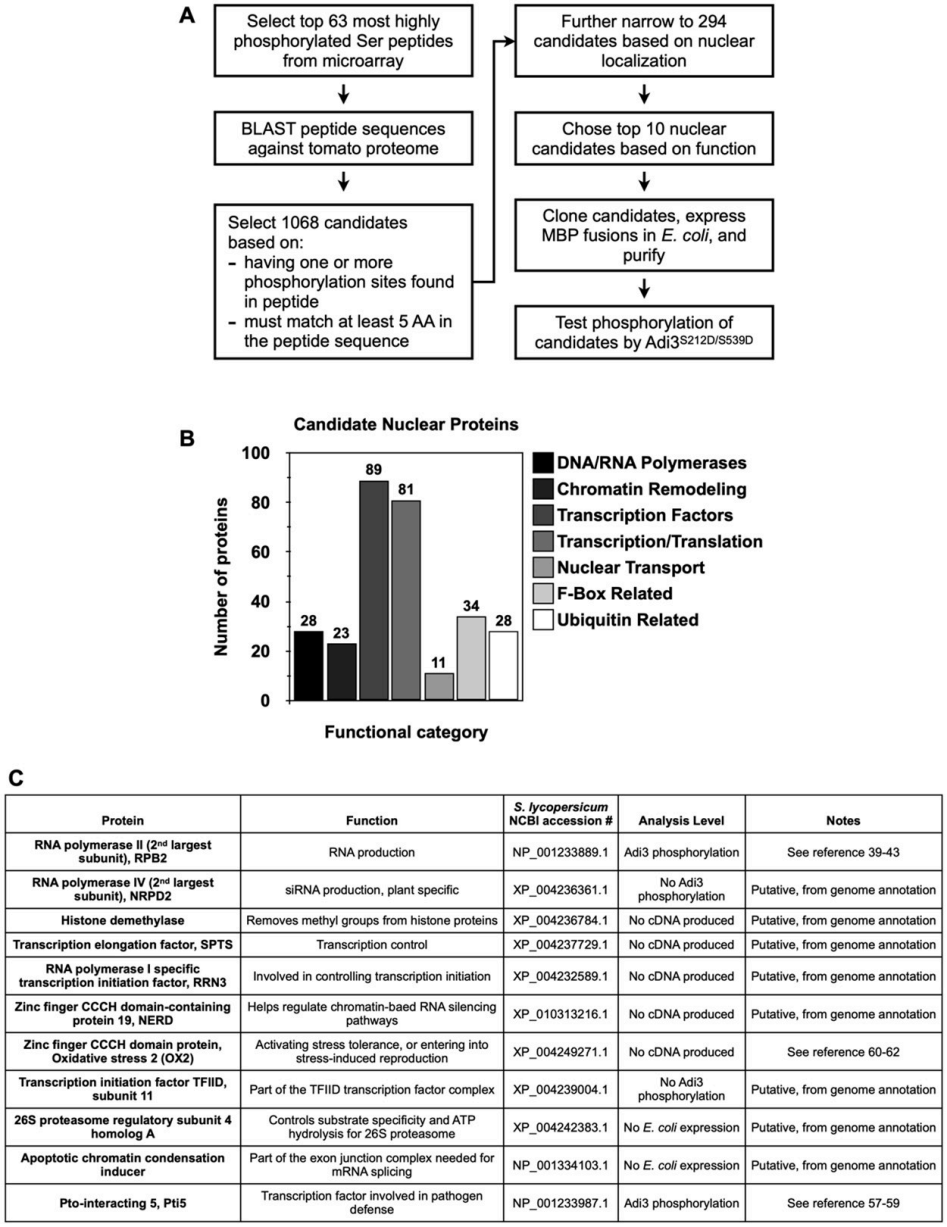
Identification of potential Adi3 nuclear substrates. (A) Bioinformatics and experimental steps followed to screen putative nuclear substrates for Adi3. (B) Categorization of 294 selected nuclear or nuclear event-related proteins identified by BLAST using the top 63 Ser peptides phosphorylated by Adi3. (C) Information for the final 11 tomato protein candidates as potential Adi3 substrates. See [31–33] for information on OX2.

We performed BLAST searches of the tomato proteome using the top 63 phosphorylated peptide sequences to identify putative nuclear substrates of Adi3. Each peptide sequence was used as a query and tomato proteins with similar sequences were identified following the steps shown in Fig 4A. Initially, the BLAST analysis identified 1,068 candidates from the top 63 peptides, and all identified proteins are listed in S1 Dataset. These candidates were selected based on two criteria: 1) each candidate must have conserved at least one of the potential phosphorylation sites in the peptide, i.e. the central Ser or additional Ser or Thr in the peptide sequence that could also be phosphorylated; and 2) each candidate much have at least 5 amino acids conserved from the phosphorylated peptide sequence. See S1 Dataset for details.

Next, these 1,068 candidates were further filtered down to 294 candidates by selecting proteins with predicted nuclear localization and/or nuclear localized functions. These 294 candidates were classified by functions (Fig 4B) and are listed in S2 Dataset. Most of the candidate proteins were identified as transcriptional and translational regulators (Fig 4B). Other functional categories included proteins involved in DNA or RNA polymerase complexes, candidates associated with chromatin remodeling, nuclear transport, and ubiquitin-related degradation (Fig 4B).

Finally, these 294 candidates were filtered to a list of ten potential candidates based on their similarities to the phosphorylated peptide sequences and function as related to the interests of our laboratory (Fig 4C). As mentioned above, the sequence of an additional 101 phosphorylated peptides past the top 63 phosphorylated peptides were also used in BLAST searches. From this screen the 92^nd^ peptide was found as a match to candidate 3 and the 139^th^ peptide was found to match candidate 2 (S4B Fig, S3 Table). An eleventh candidate, the pathogenesis defense transcription factor Pto-interacting 5 (Pti5), was also selected (Fig 4C) based on similarity to peptide 164 (S4B Fig, S3 Table).

### Phosphorylation of RPB2 and Pti5 as potential nuclear substrates for Adi3

In order to determine whether any of the eleven identified tomato proteins are real substrates for Adi3, we attempted to clone, express in *E*. *coli*, and purify all eleven candidates for testing phosphorylation by Adi3 using *in vitro* kinase assays. However, five of the cDNAs, histone demethylase, transcription elongation factor SPTS, RNA polymerase I specific transcription initiation factor (RRN3), zinc finger CCCH domain-containing protein 19 (NERD), and zinc finger CCCH domain protein oxidative stress 2 (OX2), were not able to be amplified by RT-PCR (Fig 4C). The remaining six cDNAs were able to be isolated and cloned into the pMAL-c2x vector for expression in *E*. *coli* as a maltose binding protein (MBP) fusion. Of these six cDNAs, the 26S proteasome regulatory subunit 4 homolog A and apoptotic chromatin condensation inducer were not expressible in *E*. *coli* (Fig 4C), possibly due to protein solubility issues. Finally, four cDNAs, RNA polymerase II 2^nd^ largest subunit (RPB2), RNA polymerase IV 2^nd^ largest subunit (NRPD2), transcription initiation factor TFIID subunit 11, and Pti5 (Fig 4C), were expressed and purified from *E*. *coli* as MBP fusion proteins.

The full length NRPD2 protein did not express in *E*. *coli*. Thus, a subdomain of 357 amino acids containing the two potential phosphorylated Ser residues (S6A Fig, S3 Table) was expressed, purified, and tested for Adi3 phosphorylation. The results indicate that a protein was phosphorylated in this assay, however, this protein was not at the expected size for the NRPD2 subdomain (S6B Fig). The full length TFIID subunit 11 protein was expressible in *E*. *coli* and an Adi3 kinase assay indicates it was not phosphorylated by Adi3 (S6C Fig). These results suggest neither NRPD2 or TFIID subunit 11 are kinase substrates for Adi3 and they were not studied further.

**Fig 5:**
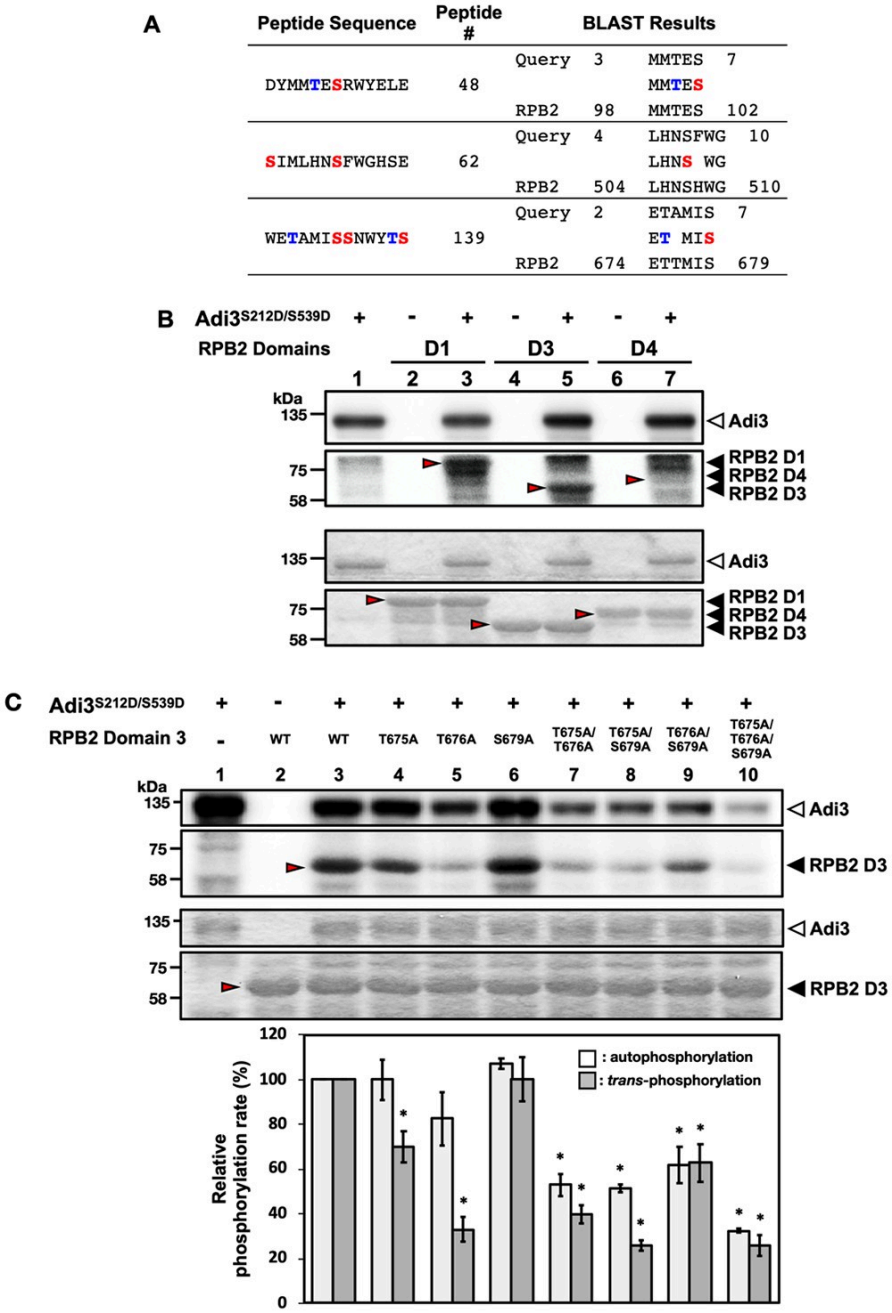
Confirmation of Adi3-mediated phosphorylation events on RPB2 as a potential substrate for Adi3. (A) BLAST results from the identification of RPB2 as a potential Adi3 substrate. RPB2 was identified by BLAST using the 48^th^, 62^th^, and 139^th^ peptide as queries. In the peptide sequence column, Ser and Thr residues highlighted in red or blue, respectively, indicate possible phosphorylation sites. Peptide # refers to the ranking of each indicated peptide used for BLAST within the top 63 peptides and the 139^th^ peptide phosphorylated by Adi3. In the BLAST results column, numbers represent amino acid positions in the peptide or RPB2 protein. (B) *in vitro* kinase activity of Adi3 toward RPB2. Three μg of each RPB2 domain protein was incubated with 1 μCi of [γ-^32^P]ATP in the presence or absence of 1 μg of Adi3^S212D/S595D^. Red arrows indicate the expected position of RPB2 domain proteins. Top and bottom panels show the phosphorimage and Coomassie stained gel, respectively. Experiments were repeated three times with similar results. (C) Adi3 phosphorylates Thr675 and Thr676 of RPB2 D3. The indicated RPB2 D3 Thr or Ser residues were mutated to Ala individually or in combination and tested for Adi3-mediated phosphorylation using *in vitro* kinase assays. The assay was conducted as described in B. Quantification of the auto- and *trans*-phosphorylation activities of Adi3 were from three independent assays. Top and bottom panels indicate the phosphorimage and Coomassie stained gel, respectively. Asterisks indicate significantly decreased (*) auto- and *trans*-phosphorylation activity of Adi3 compared to RPB2 D3^WT^ (Student’ *t* test, *P* < 0.05). Error bars represent standard error.

For RPB2, the protein was not able to be expressed as a full protein likely due to its large molecular weight, 135.1 kDa plus 42 kDa for MBP to give a 177.1 kDa protein. Thus, RPB2 was divided into 4 domains of roughly equal molecular weight for separate production in *E*. *coli* (S7A and S7B Fig). Initially, RPB2 was identified by the 48^th^ and 62^nd^ peptides in the Ser-peptide microarray analysis (Fig 5A, S3 Table). Additionally, when the 139^th^ peptide (S4B Fig, S2 Table, S1 Dataset) was used as a BLAST query, it identified RPB2 as a candidate (Fig 5A, S3 Table). Therefore, the RPB2 domain 1 (D1), domain 2 (D2), and domain 3 (D3) contain potential phosphorylation sites identified by the 48^th^, 62^nd^, and 139^th^ peptides, respectively (S7B Fig). The RPB2 domain 4 (D4) was also analyzed for Adi3 phosphorylation even though it did not contain a predicted Adi3 phosphorylation site from the peptide analysis. When these four RPB2 domains were expressed in *E*. *coli* as MBP fusions, the D2 protein was not expressed and was not further analyzed. The D1, D3, and D4 domains were expressed and purified as MBP fusions, and only D1 and D3 were found to be phosphorylated by Adi3 in *in vitro* kinase assays (Fig 5B, lanes 3, 5). Phosphorylation of a protein in the RPB2 D4 sample was seen, however, this phosphorylated protein was not at the expected size of the D4 protein (Fig 5B, lane 7). To analyze the possibility of RPB2 domain phosphorylation by contamination with other kinases derived from *E*. *coli*, each RPB2 domain was incubated with ^32^P-ATP in the absence of Adi3. This analysis showed that none of the RPB2 domain proteins were phosphorylated (Fig 5B, lanes 2, 4, 6). These data confirm the ability of Adi3 to phosphorylate RPB2.

Adi3-mediated phosphorylation of Pti5 was also tested using *in vitro* kinase assays. As indicated above, Pti5 was identified as a potential Adi3 substrate by using the 164^th^ peptide in the BLAST search (S8A Fig). Pti5 is a short protein of 161 amino acids and the predicted phosphorylation site is found at position 16 (S8B Fig). For the expression of Pti5 in *E*. *coli* as an MBP fusion, the protein purified as a doublet of proteins (S8C Fig, bottom panel), and both of these proteins were phosphorylated by Adi3 (S8C Fig). The Pti5^S16A^ mutant was tested for a loss of phosphorylation by Adi3, but there was no difference in the phosphorylation level of Pti5^S16A^ as compared to Pti5^WT^ (S8D Fig), indicating Adi3 phosphorylates Pti5 at one of the 14 other Ser residues or possibly one of the 6 Thr residues.

### Identification of the RPB2 residues phosphorylated by Adi3

Alignment of the phosphorylated Ser peptides that matched RPB2 identified Ser 102 in D1, Ser507 in D2, and Ser 679 in D3 as potential Adi3 phosphorylation sites based on the central Ser in the phosphorylated peptides (Fig 5A; S7A and S7B Fig). In the RPB2 D1 and D3 regions aligning to the peptides additional Thr residues are found that could be phosphorylated by Adi3; Thr100 in D1, and Thr675 and Thr676 in D3 (Fig 5A; S7A and S7B Fig). Thus, these Ser and Thr amino acids were mutated to Ala individually and in combinations, and the proteins tested for loss of phosphorylation by Adi3 using *in vitro* kinase assays. Since RPB2 D2 was not expressible in *E*. *coli* and phosphorylation of D4 was not seen (Fig 5B, lane 7), only the RPB2 D1 Thr100/Ser102 and the D3 Thr675/676 and Ser679 Ala mutants were tested for loss of Adi3-mediatd phosphorylation. When the RPB2 D1^T100A/S102A^ protein was tested in *in vitro* kinase assays, Adi3 showed kinase activity on D1^T100A/S102A^ similar to D1^WT^ (S9 Fig, lanes 4, 5), suggesting Adi3 does not phosphorylate either of these residues or additional Ser or Thr residues are phosphorylated in addition to Thr100 and Ser102. Phosphorylation of the D3^T675A^, D3^T676A^, and D3^S679A^ mutants by Adi3 showed that the D3^T675A^ and D3^T676A^ mutants, but not D3^S679A^, had reduced phosphorylation levels compared to D3^WT^ (Fig 5C, lanes 3, 4, 5, 6). This suggests Thr675 and Thr676 are Adi3 phosphorylation sites on RPB2. However, neither of the D3^T675A^ or D3^T676A^ mutants completely eliminated phosphorylation by Adi3, indicating there are additional Adi3 phosphorylation sites on RPB2. Interestingly, when Adi3 was tested for phosphorylation activity against combinations of RPB2 D3 T675A, T676A, and S679A double and triple mutants, Adi3 showed a reduction in D3 phosphorylation, but also a reduction in Adi3 autophosphorylation activity compared to incubation with the RPB2 D3^WT^ or D3 single mutants (Fig 5C, lanes 7, 8, 9, 10).

## Discussion

### Adi3 has promiscuous kinase activity

In the peptide microarray analysis, Adi3, as a Ser/Thr protein kinase showed promiscuous kinase activity toward diverse Ser and Thr peptide sequences (Fig 2A, S3 Fig, S1 Dataset). However, more of the Ser peptides were phosphorylated, 22.5% of all Ser peptides, as compared to the Thr peptides, 8.3% of the Thr peptides (S2 Fig). These results may suggest Adi3 has a wide range of *in vivo* phosphorylation substrates, which is comparable to mammalian PKB. We have shown that Adi3 is a functional homologue to PKB [18–21, 23, 24, 28, 34] as both of these Ser/Thr protein kinases function in the suppression of PCD. Since PKB was first discovered [35], about 300 substrates of PKB have been reported to date [36]. PKB directly phosphorylates multiple targets involved in diverse cellular functions such as cell proliferation, growth, and survival including PCD regulation, transcription, and glucose metabolism [37–39]. Due to the diversity in PKB substrates, an authentic PKB phosphorylation consensus motif is not well defined. Nevertheless, a primary PKB recognition motif of R-X-R-X-X-S/T-Ф for phosphorylation has been determined [4], where X represents any amino acid and Ф denotes hydrophobic residues containing a large side chain such as Phe and Trp.

Similarly, in this study since Adi3 phosphorylated a large number of peptides a clear phosphorylation consensus sequence could not be identified. But, several important preferences can be identified for Adi3 phosphorylation sites. When considering the top 63 phosphorylated Ser peptides in a position-independent manner, Adi3 showed preference for peptides containing large hydrophobic amino acids such as Trp, Tyr, and Leu and to a lesser extent Pro, Val, and Phe, while the positively charged residues Arg and Lys were not favored (Fig 3A). Interestingly, when considering the position-dependent analysis the positively charged amino acid His was frequently found in the sequence of the top 10 phosphorylated peptides (Fig 3C). In the position-dependent analysis for the top 10 phosphorylated Thr peptides, several Tyr residues were conserved downstream of the central Thr, and Trp was conserved upstream of the Thr residue (Fig 3D). Taken together, Adi3 showed preference for aromatic and cyclic amino acids in both Ser and Thr phosphorylated peptides, however, due to the promiscuous activity of Adi3 for diverse peptides, a bona fide phosphorylation motif was not identified, similar to PKB.

### Putative nuclear substrates for Adi3

Despite the significant role of Adi3 in suppression of plant programmed cell death, little is known about its downstream substrates. To understand a mechanism of Adi3 CDS in the nucleus, several approaches have been attempted to screen for Adi3 phosphorylation substrates [20, 21, 24, 34]. However, Gal83, identified indirectly through a yeast two-hybrid screen, is the only validated Adi3 phosphorylation substrate [20]. This yeast two-hybrid screen identified other Adi3 interactors that are not phosphorylation substrates, such as the E3 ubiquitin ligase AdBiL [21] and the autophagy protein Atg8h [34].

In the studies presented here, the 2^nd^ largest subunit of RNA polymerase II, RPB2, was identified as a potential Adi3 phosphorylation substrate by three different phosphorylated peptides from the Ser-peptide microarray, the 48^th^, 62^nd^, and 139^th^ peptides (Fig 5A), and we showed Adi3 can phosphorylate RPB2 on Thr675 and Thr676 (Fig 5B and 5C). In these kinase assays we noticed the RPB2 D3 double and triple Ala mutations of the possible phosphorylation sites had a negative effect on Adi3 autophosphorylation as well as *trans*-phosphorylation (Fig 5C). This alteration in Adi3 phosphorylation activity may be explained by the RPB2 D3 double and triple Ala mutants causing a conformational change, which alters interaction with Adi3 and consequently compromises Adi3 auto- and *trans*-phosphorylation activity. It has been reported that a mutated substrate can affect the autophosphorylation activity of its protein kinase [40]. In mammals, protein kinase R (PKR) phosphorylates eukaryotic translation initiation factor 2α (eIF2α) on Ser51 [40, 41]. In an effort to understand the function in PKR-mediated phosphorylation of the eIF2α flexible loop containing Ser51, various eIF2α Ala mutants were generated at and immediately upstream and downstream of the Ser51 site for analysis of PKR phosphorylation. In the presence of both the eIF2α^S51A^ and the upstream and downstream Ala mutants PKR showed alterations in autophosphorylation levels while the eIF2α mutants were not phosphorylated or had reduced PKR phosphorylation levels [40]. This suggests alterations in kinase autophosphorylation in the presence of mutated substrates may be a wider phenomenon.

Phosphorylation of RPB2 Thr675 and Thr676 by Adi3 is well supported since the single Ala mutants for these residues showed a decrease in Adi3 phosphorylation without a loss in Adi3 autophosphorylation (Fig 5C). Interestingly, while these data (Fig 2D; S2A and S2B Fig) and past studies [20] show Adi3 favors phosphorylation on Ser residues, Thr phosphorylation on RPB2 by Adi3 was identified. Thus, future studies should not discount the possibility of Thr substrate phosphorylation by Adi3.

In eukaryotes, the RNA polymerase II (RPB) complex is responsible for transcription of mRNA from protein-coding genes as well as small RNAs, and the complex consists of 10 to 14 subunits [42, 43]. Of these subunits, RPB2 is the most highly conserved among eukaryotic species [44], and in humans, RPB2 is dispensable for transcription initiation and elongation steps [45]. Recently, in yeast (*Saccharomyces cerevisiae*), it has been demonstrated that RPB2 regulates termination of transcription via polyadenylation [46–48]. Furthermore, a function for RPB2 in plant developmental processes was recently reported [49].

Phosphorylation plays a role in the regulation of the RPB complex [50]. RPB contains heptapeptide repeats of Y-S-P-T-S-P-S on the carboxyl-terminal domain (CTD) of the largest subunit RPB1 [51]. Alterations in the phosphorylation levels of the heptapeptide repeat control the RPB transcription cycle and recruits other accessory proteins required for the elongation and termination phases [52, 53]. Studies on phosphorylation-mediated regulation of other RPB subunits have not been reported to date, although RPB2 and RPB4 phosphorylation events were confirmed in extracts of ^32^P-labelled yeast cells [54]. Based on these studies and our Adi3 phosphorylation results, we suggest it may be necessary to analyze a function for phosphorylation events on RPB2. Particularly, the *in vivo* phosphorylation of RPB2 by Adi3 and a possible function for this event will need to be confirmed in the future.

In the interaction between the host tomato and the *Pst* pathogen, the tomato Pto kinase is a resistance protein [55] which, in concert with the Prf nucleotide-binding leucine-rich repeat protein [56], specifically recognizes and forms a complex with the *Pst*-derived effector protein AvrPto [57] in order to initiate a host resistance response to *Pst* [58]. In an effort to comprehend Pto-mediated host defense against *Pst*, several Pto-interacting (Pti) proteins were previously isolated and characterized [59, 60]. Pti5 is an ethylene response element-binding protein-like transcription factor [61]. Formation of the AvrPto/Pto complex enhances Pti5 expression, which consequentially stimulates expression of pathogen-induced genes such as, *GluB* and *catalase* encoding β-1,3-glucanases and catalase, respectively [62]. Therefore, Pti5 is responsible for positive regulation for plant defense responses.

Despite the role of Pti5 in host disease resistance, regulatory mechanisms controlling Pti5 function, particularly the possibility of phosphorylation, has not been determined. Our current studies may suggest Adi3-mediated Pti5 regulation via a phosphorylation event. Given that in the absence of *Pst* Adi3 functions in the nucleus to suppress PCD [19, 23], Adi3 phosphorylation may inhibit Pti5 function. In the defense response to *Pst*, Adi3 interacts with the Pto/AvrPto complex preventing Adi3 nuclear entry leading to a loss of Adi3 CDS [23]. This may prevent Adi3 phosphorylation of Pti5 and possibly activate Pti5 to stimulate expression of defense-related genes to regulate *Pst* infection. However, to support this hypothesis the identification of the Adi3 phosphorylated residue(s) on Pti5 and its effect on Pti5 activity is required.

In conclusion, the diverse phosphorylated peptides identified by microarray peptide phosphorylation analysis were utilized to profile substrates of the Adi3 kinase. Furthermore, the potential Adi3 phosphorylation candidates found in this study may provide a starting point to understand the mechanism for Adi3 CDS as well as other cellular functions.

## Supporting information

S1 Fig Test of Adi3S212D/S539D kinase activity and phosphorylated Thr-peptide microarray. (PDF)

S2 Fig Position of all peptides phosphorylated by Adi3 on the Ser- and Thr-peptide microarrays. (PDF)

S3 Fig Mapping and comparison of Adi3 phosphorylated peptides on the Ser- and Thr-peptide microarray chips. (PDF)

S4 Fig Distribution of mean signal intensities for phosphorylated peptides and position of the top 63 Adi3 phosphorylated Ser peptides. (PDF)

S5 Fig Sequence logos based on different numbers of Adi3-phosphorylated peptides. (PDF)

S6 Fig Adi3 does not phosphorylate NRPD2 or TFIID. (PDF)

S7 Fig Protein domains of RNA polymerase II, second largest subunit (RPB2). (PDF)

S8 Fig Identification and analysis of Pti5 as a potential Adi3 substrate. (PDF)

S9 Fig Adi3 does not phosphorylate RPB2 domain 1 (D1) at T100 or S102. (PDF)

S1 Table Primers used in this study. (PDF)

S2 Table Sequence of the top 63 phosphorylated Ser peptides. (PDF)

S3 Table Final 11 potential Adi3 phosphorylation candidates. (PDF)

S1 Dataset (XLSX)

S2 Dataset (XLSX)

S1 Raw images (PDF)

## References

[1] Greenberg JT. Programmed Cell Death in Plant-Pathogen Interactions. Annu Rev Plant Physiol Plant Mol Biol. 1997;48:525–45. 10.1146/annurev.arplant.48.1.525 . 15012273

[2] Hochman A. Programmed cell death in prokaryotes. Critical reviews in microbiology. 1997;23(3):207–14. Epub 1997/01/01. 10.3109/10408419709115136 . 9347220

[3] Manning BD, Cantley LC. AKT/PKB signaling: navigating downstream. Cell. 2007;129(7):1261–74. Epub 2007/07/03. 10.1016/j.cell.2007.06.009 . 17604717 PMC2756685

[4] Manning BD, Toker A. AKT/PKB Signaling: Navigating the Network. Cell. 2017;169(3):381–405. Epub 2017/04/22. 10.1016/j.cell.2017.04.001 28431241 PMC5546324

[5] Brunet A, Bonni A, Zigmond MJ, Lin MZ, Juo P, Hu LS, et al Akt promotes cell survival by phosphorylating and inhibiting a Forkhead transcription factor. Cell. 1999;96(6):857–68. 10.1016/s0092-8674(00)80595-4 . 10102273

[6] Morel C, Carlson SM, White FM, Davis RJ. Mcl-1 integrates the opposing actions of signaling pathways that mediate survival and apoptosis. Molecular and cellular biology. 2009;29(14):3845–52. Epub 2009/05/13. 10.1128/mcb.00279-09 19433446 PMC2704749

[7] Liu G, Bi Y, Wang R, Shen B, Zhang Y, Yang H, et al Kinase AKT1 negatively controls neutrophil recruitment and function in mice. Journal of immunology (Baltimore, Md: 1950). 2013;191(5):2680–90. Epub 2013/08/02. 10.4049/jimmunol.1300736 . 23904165

[8] Friant S, Lombardi R, Schmelzle T, Hall MN, Riezman H. Sphingoid base signaling via Pkh kinases is required for endocytosis in yeast. EMBO J. 2001;20(23):6783–92. Epub 2001/12/01. 10.1093/emboj/20.23.6783 11726514 PMC125749

[9] Jacinto E, Hall MN. Tor signalling in bugs, brain and brawn. Nature reviews Molecular cell biology. 2003;4(2):117–26. Epub 2003/02/04. 10.1038/nrm1018 . 12563289

[10] Kozma SC, Thomas G. Regulation of cell size in growth, development and human disease: PI3K, PKB and S6K. BioEssays: news and reviews in molecular, cellular and developmental biology. 2002;24(1):65–71. Epub 2002/01/10. 10.1002/bies.10031 . 11782951

[11] Zhang SH, Lawton MA, Hunter T, Lamb CJ. atpk1, a novel ribosomal protein kinase gene from Arabidopsis. I. Isolation, characterization, and expression. The Journal of biological chemistry. 1994;269(26):17586–92. Epub 1994/07/01. . 7912697

[12] Pearce LR, Komander D, Alessi DR. The nuts and bolts of AGC protein kinases. Nature reviews Molecular cell biology. 2010;11(1):9–22. Epub 2009/12/23. 10.1038/nrm2822 . 20027184

[13] Lam E. Controlled cell death, plant survival and development. Nature reviews Molecular cell biology. 2004;5(4):305–15. Epub 2004/04/09. 10.1038/nrm1358 . 15071555

[14] Jones JD, Dangl JL. The plant immune system. Nature. 2006;444(7117):323–9. Epub 2006/11/17. 10.1038/nature05286 . 17108957

[15] Nurnberger T, Brunner F, Kemmerling B, Piater L. Innate immunity in plants and animals: striking similarities and obvious differences. Immunol Rev. 2004;198:249–66. Epub 2004/06/18. 10.1111/j.0105-2896.2004.0119.x . 15199967

[16] Van der Biezen EA, Jones JDG. Plant disease-resistance proteins and the gene-for-gene concept. Trends Biochem Sci. 1998;23(12):454–6. 9868361 10.1016/s0968-0004(98)01311-5

[17] Reape TJ, McCabe PF. Apoptotic-like programmed cell death in plants. The New phytologist. 2008;180(1):13–26. Epub 2008/07/18. 10.1111/j.1469-8137.2008.02549.x . 18631291

[18] Devarenne TP, Ekengren SK, Pedley KF, Martin GB. Adi3 is a Pdk1-interacting AGC kinase that negatively regulates plant cell death. Embo j. 2006;25(1):255–65. Epub 2005/12/20. 10.1038/sj.emboj.7600910 16362044 PMC1356353

[19] Ek-Ramos MJ, Avila J, Cheng C, Martin GB, Devarenne TP. The T-loop extension of the tomato protein kinase AvrPto-dependent Pto-interacting protein 3 (Adi3) directs nuclear localization for suppression of plant cell death. The Journal of biological chemistry. 2010;285(23):17584–94. Epub 2010/04/08. 10.1074/jbc.M110.117416 20371603 PMC2878523

[20] Avila J, Gregory OG, Su D, Deeter TA, Chen S, Silva-Sanchez C, et al The beta-subunit of the SnRK1 complex is phosphorylated by the plant cell death suppressor Adi3. Plant physiology. 2012;159(3):1277–90. Epub 2012/05/11. 10.1104/pp.112.198432 22573803 PMC3387709

[21] Avila J, Devarenne TP. Ubiquitination of the tomato cell death suppressor Adi3 by the RING E3 ubiquitin ligase AdBiL. Biochemical and biophysical research communications. 2013;430(1):119–24. Epub 2012/11/28. 10.1016/j.bbrc.2012.11.043 . 23178567

[22] Gray JW, Nelson Dittrich AC, Chen S, Avila J, Giavalisco P, Devarenne TP. Two Pdk1 phosphorylation sites on the plant cell death suppressor Adi3 contribute to substrate phosphorylation. Biochim Biophys Acta. 2013;1834(6):1099–106. Epub 2013/03/20. 10.1016/j.bbapap.2013.03.006 . 23507047 PMC4301410

[23] Ek-Ramos MJ, Avila J, Nelson Dittrich AC, Su D, Gray JW, Devarenne TP. The tomato cell death suppressor Adi3 is restricted to the endosomal system in response to the Pseudomonas syringae effector protein AvrPto. PloS one. 2014;9(10):e110807 Epub 2014/10/29. 10.1371/journal.pone.0110807 25350368 PMC4211712

[24] Dittrich AC, Devarenne TP. An ATP analog-sensitive version of the tomato cell death suppressor protein kinase Adi3 for use in substrate identification. Biochimica et biophysica acta. 2012;1824(2):269–73. Epub 2011/10/27. 10.1016/j.bbapap.2011.10.004 . 22027266

[25] Devarenne TP, Martin GB. Manipulation of plant programmed cell death pathways during plant-pathogen interactions. Plant signaling & behavior. 2007;2(3):188–9. Epub 2007/05/01. 10.4161/psb.2.3.4150 19704693 PMC2634054

[26] Halford NG, Hey SJ. Snf1-related protein kinases (SnRKs) act within an intricate network that links metabolic and stress signalling in plants. Biochem J. 2009;419(2):247–59. Epub 2009/03/25. 10.1042/BJ20082408 . 19309312

[27] Dominy CN, Andrews DW. Site-Directed Mutagenesis by Inverse PCR In: Casali N, Preston A, editors. E coli Plasmid Vectors: Methods and Applications. Totowa, NJ: Humana Press; 2003 p. 209–23. 10.1385/1-59259-409-3:209 12904664

[28] Gray JW, Nelson Dittrich AC, Chen S, Avila J, Giavalisco P, Devarenne TP. Two Pdk1 phosphorylation sites on the plant cell death suppressor Adi3 contribute to substrate phosphorylation. Biochimica et biophysica acta. 2013;1834(6):1099–106. Epub 2013/03/20. 10.1016/j.bbapap.2013.03.006 23507047 PMC4301410

[29] Smolka B, Lukac R, Plataniotis KN, Venetsanopoulos AN. Application of kernel density estimation for color image filtering: SPIE; 2003.

[30] Crooks GE, Hon G, Chandonia JM, Brenner SE. WebLogo: a sequence logo generator. Genome research. 2004;14(6):1188–90. Epub 2004/06/03. 10.1101/gr.849004 15173120 PMC419797

[31] Sun J, Jiang H, Xu Y, Li H, Wu X, Xie Q, et al The CCCH-type zinc finger proteins AtSZF1 and AtSZF2 regulate salt stress responses in Arabidopsis. Plant & cell physiology. 2007;48(8):1148–58. Epub 2007/07/03. 10.1093/pcp/pcm088 . 17609218

[32] Blanvillain R, Wei S, Wei P, Kim JH, Ow DW. Stress tolerance to stress escape in plants: role of the OXS2 zinc-finger transcription factor family. The EMBO journal. 2011;30(18):3812–22. 10.1038/emboj.2011.270 . 21829164 PMC3173794

[33] He L, Ma X, Li Z, Jiao Z, Li Y, Ow DW. Maize OXIDATIVE STRESS2 Homologs Enhance Cadmium Tolerance in Arabidopsis through Activation of a Putative SAM-Dependent Methyltransferase Gene. Plant physiology. 2016;171(3):1675–85. Epub 2016/05/17. 10.1104/pp.16.00220 . 27208260 PMC4936553

[34] Devarenne TP. The plant cell death suppressor Adi3 interacts with the autophagic protein Atg8h. Biochem Biophys Res Commun. 2011;412(4):699–703. Epub 2011/08/27. 10.1016/j.bbrc.2011.08.031 . 21867679

[35] Staal SP. Molecular cloning of the akt oncogene and its human homologues AKT1 and AKT2: amplification of AKT1 in a primary human gastric adenocarcinoma. Proc Natl Acad Sci U S A. 1987;84(14):5034–7. 10.1073/pnas.84.14.5034 . 3037531 PMC305241

[36] Yudushkin I. Getting the Akt Together: Guiding Intracellular Akt Activity by PI3K. Biomolecules. 2019;9(2). Epub 2019/02/20. 10.3390/biom9020067 . 30781447 PMC6406913

[37] Bago R, Sommer E, Castel P, Crafter C, Bailey FP, Shpiro N, et al The hVps34-SGK3 pathway alleviates sustained PI3K/Akt inhibition by stimulating mTORC1 and tumour growth. Embo j. 2016;35(20):2263 Epub 2016/11/01. 10.15252/embj.201670010 27798145 PMC5069549

[38] Hirai H, Sootome H, Nakatsuru Y, Miyama K, Taguchi S, Tsujioka K, et al MK-2206, an allosteric Akt inhibitor, enhances antitumor efficacy by standard chemotherapeutic agents or molecular targeted drugs in vitro and in vivo. Molecular cancer therapeutics. 2010;9(7):1956–67. Epub 2010/06/24. 10.1158/1535-7163.Mct-09-1012 . 20571069

[39] Hawley SA, Ross FA, Gowans GJ, Tibarewal P, Leslie NR, Hardie DG. Phosphorylation by Akt within the ST loop of AMPK-alpha1 down-regulates its activation in tumour cells. Biochem J. 2014;459(2):275–87. Epub 2014/01/29. 10.1042/bj20131344 24467442 PMC4052680

[40] Uppala JK, Ghosh C, Sathe L, Dey M. Phosphorylation of translation initiation factor eIF2alpha at Ser51 depends on site- and context-specific information. FEBS Lett. 2018;592(18):3116–25. Epub 2018/08/03. 10.1002/1873-3468.13214 30070006 PMC6167009

[41] Hinnebusch AG. The scanning mechanism of eukaryotic translation initiation. Annual review of biochemistry. 2014;83:779–812. Epub 2014/02/07. 10.1146/annurev-biochem-060713-035802 . 24499181

[42] Trinh V, Langelier MF, Archambault J, Coulombe B. Structural perspective on mutations affecting the function of multisubunit RNA polymerases. Microbiology and molecular biology reviews: MMBR. 2006;70(1):12–36. Epub 2006/03/10. 16524917 10.1128/MMBR.70.1.12-36.2006 PMC1393249

[43] Young RA. RNA polymerase II. Annual review of biochemistry. 1991;60:689–715. Epub 1991/01/01. 10.1146/annurev.bi.60.070191.003353 . 1883205

[44] Langelier MF, Baali D, Trinh V, Greenblatt J, Archambault J, Coulombe B. The highly conserved glutamic acid 791 of Rpb2 is involved in the binding of NTP and Mg(B) in the active center of human RNA polymerase II. Nucleic acids research. 2005;33(8):2629–39. Epub 2005/05/12. 10.1093/nar/gki570 15886393 PMC1092279

[45] Palangat M, Grass JA, Langelier MF, Coulombe B, Landick R. The RPB2 flap loop of human RNA polymerase II is dispensable for transcription initiation and elongation. Molecular and cellular biology. 2011;31(16):3312–25. Epub 2011/06/15. 10.1128/mcb.05318-11 21670157 PMC3147802

[46] Kubicek CE, Chisholm RD, Takayama S, Hawley DK. RNA polymerase II mutations conferring defects in poly(A) site cleavage and termination in Saccharomyces cerevisiae. G3 (Bethesda, Md). 2013;3(2):167–80. Epub 2013/02/08. 10.1534/g3.112.004531 23390594 PMC3564978

[47] Cui Y, Denis CL. In vivo evidence that defects in the transcriptional elongation factors RPB2, TFIIS, and SPT5 enhance upstream poly(A) site utilization. Molecular and cellular biology. 2003;23(21):7887–901. Epub 2003/10/16. 10.1128/MCB.23.21.7887-7901.2003 14560031 PMC207619

[48] Kaplan CD, Holland MJ, Winston F. Interaction between transcription elongation factors and mRNA 3’-end formation at the Saccharomyces cerevisiae GAL10-GAL7 locus. The Journal of biological chemistry. 2005;280(2):913–22. Epub 2004/11/09. 10.1074/jbc.M411108200 . 15531585

[49] Chen L, Guan L, Qian P, Xu F, Wu Z, Wu Y, et al NRPB3, the third largest subunit of RNA polymerase II, is essential for stomatal patterning and differentiation in Arabidopsis. Development (Cambridge, England). 2016;143(9):1600–11. Epub 2016/03/19. 10.1242/dev.129098 26989174 PMC4909857

[50] Buratowski S. Connections between mRNA 3’ end processing and transcription termination. Current opinion in cell biology. 2005;17(3):257–61. Epub 2005/05/20. 10.1016/j.ceb.2005.04.003 . 15901494

[51] Jeronimo C, Collin P, Robert F. The RNA Polymerase II CTD: The Increasing Complexity of a Low-Complexity Protein Domain. Journal of molecular biology. 2016;428(12):2607–22. Epub 2016/02/16. 10.1016/j.jmb.2016.02.006 . 26876604

[52] Hajheidari M, Koncz C, Eick D. Emerging roles for RNA polymerase II CTD in Arabidopsis. Trends in plant science. 2013;18(11):633–43. Epub 2013/08/06. 10.1016/j.tplants.2013.07.001 . 23910452

[53] Antosz W, Pfab A, Ehrnsberger HF, Holzinger P, Kollen K, Mortensen SA, et al The Composition of the Arabidopsis RNA Polymerase II Transcript Elongation Complex Reveals the Interplay between Elongation and mRNA Processing Factors. The Plant cell. 2017;29(4):854–70. Epub 2017/03/30. 10.1105/tpc.16.00735 28351991 PMC5435424

[54] Kolodziej PA, Woychik N, Liao SM, Young RA. RNA polymerase II subunit composition, stoichiometry, and phosphorylation. Molecular and cellular biology. 1990;10(5):1915–20. Epub 1990/05/01. 10.1128/mcb.10.5.1915 2183013 PMC360537

[55] Loh YT, Martin GB. The Pto bacterial resistance gene and the Fen insecticide sensitivity gene encode functional protein kinases with serine/threonine specificity. Plant physiology. 1995;108(4):1735–9. Epub 1995/08/01. 10.1104/pp.108.4.1735 7659757 PMC157555

[56] Salmeron JM, Oldroyd GE, Rommens CM, Scofield SR, Kim HS, Lavelle DT, et al Tomato *Prf* is a member of the leucine-rich repeat class of plant disease resistance genes and lies embedded within the *Pto* kinase gene cluster. Cell. 1996;86(1):123–33. Epub 1996/07/12. 10.1016/s0092-8674(00)80083-5 . 8689679

[57] Salmeron JM, Staskawicz BJ. Molecular characterization and hrp dependence of the avirulence gene avrPto from Pseudomonas syringae pv. tomato [corrected]. Molecular & general genetics: MGG. 1993;239(1–2):6–16. Epub 1993/05/01. 10.1007/BF00281595 . 8510663

[58] Frederick RD, Thilmony RL, Sessa G, Martin GB. Recognition specificity for the bacterial avirulence protein AvrPto is determined by Thr-204 in the activation loop of the tomato Pto kinase. Mol Cell. 1998;2(2):241–5. 9734361 10.1016/s1097-2765(00)80134-3

[59] Zhou J, Tang X, Martin GB. The Pto kinase conferring resistance to tomato bacterial speck disease interacts with proteins that bind a cis-element of pathogenesis-related genes. Embo j. 1997;16(11):3207–18. Epub 1997/06/02. 10.1093/emboj/16.11.3207 9214637 PMC1169938

[60] Gu YQ, Wildermuth MC, Chakravarthy S, Loh YT, Yang C, He X, et al Tomato transcription factors pti4, pti5, and pti6 activate defense responses when expressed in Arabidopsis. The Plant cell. 2002;14(4):817–31. Epub 2002/04/24. 10.1105/tpc.000794 11971137 PMC150684

[61] Thara VK, Tang X, Gu YQ, Martin GB, Zhou JM. *Pseudomonas syringae* pv *tomato* induces the expression of tomato EREBP-like genes *Pti4* and *Pti5* independent of ethylene, salicylate and jasmonate. Plant J. 1999;20(4):475–83. 10607299 10.1046/j.1365-313x.1999.00619.x

[62] He P, Warren RF, Zhao T, Shan L, Zhu L, Tang X, et al Overexpression of Pti5 in tomato potentiates pathogen-induced defense gene expression and enhances disease resistance to Pseudomonas syringae pv. tomato. Molecular plant-microbe interactions: MPMI. 2001;14(12):1453–7. Epub 2002/01/05. 10.1094/mpmi.2001.14.12.1453 . 11768541

