# Supplementary material for: Screening for potential nuclear substrates for the plant cell death suppressor kinase Adi3 using peptide microarrays
Source: PLoS One. 2020 Jun 2;15(6):e0234011. doi: 10.1371/journal.pone.0234011 (PMC7266335; doi:10.1371/journal.pone.0234011)
Supplement: S1 Fig — (PDF) [file pone.0234011.s001.pdf]

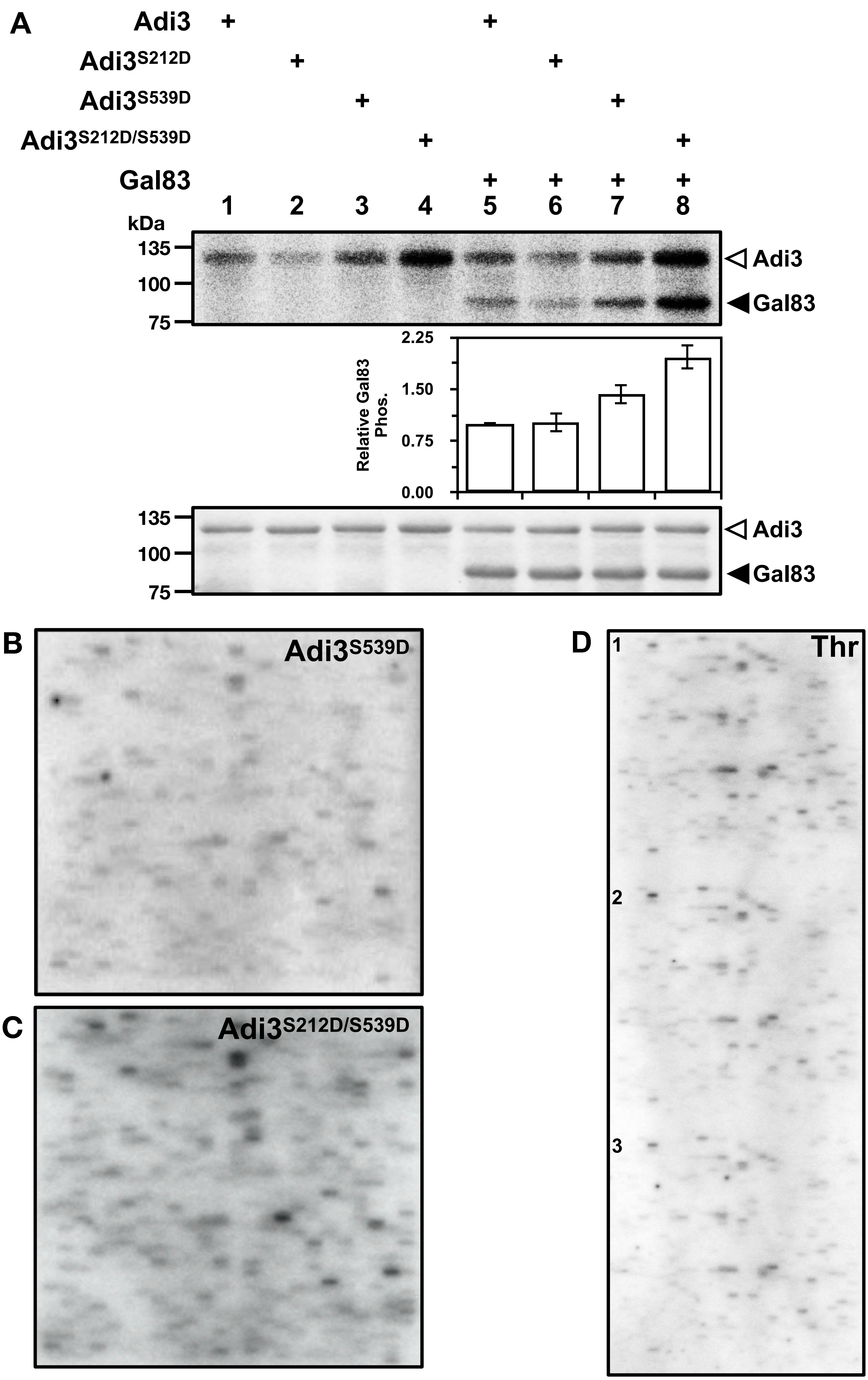

S1 Fig. Test of Adi3$^{S212D/S539D}$ kinase activity and phosphorylated Thr-peptide microarray. (A) Wild-type, single (S212D or S539D), and double (S212D/S539D) phosphomimetic mutants of Adi3 were incubated with [γ-^{32}P]ATP in the absence or presence of Gal83 in an *in vitro* kinase assay. Top panel, phosphorimage; middle panel, quantification of phosphorylated Gal83; bottom panels, Coomassie stained-gel. In the middle panel, error bars indicate standard error from three independent experiments. In B, C, phosphorimages of one subarray of a Ser-peptide microarray chip incubated with (B) Adi3^{S539D} or (C) Adi3$^{S212D/S539D}$. (D) Phosphorimage of the whole Thr-peptide microarray chip incubated with [γ-^{32}P]ATP in the presence of Adi3$^{S212D/S539D}$. Numbers represent each subarray region. Image shown is representative of 4 Thr-peptide chips phosphorylated by Adi3$^{S212D/S539D}$.
