# Supplementary material for: Screening for potential nuclear substrates for the plant cell death suppressor kinase Adi3 using peptide microarrays
Source: PLoS One. 2020 Jun 2;15(6):e0234011. doi: 10.1371/journal.pone.0234011 (PMC7266335; doi:10.1371/journal.pone.0234011)
Supplement: S2 Fig — (PDF) [file pone.0234011.s002.pdf]

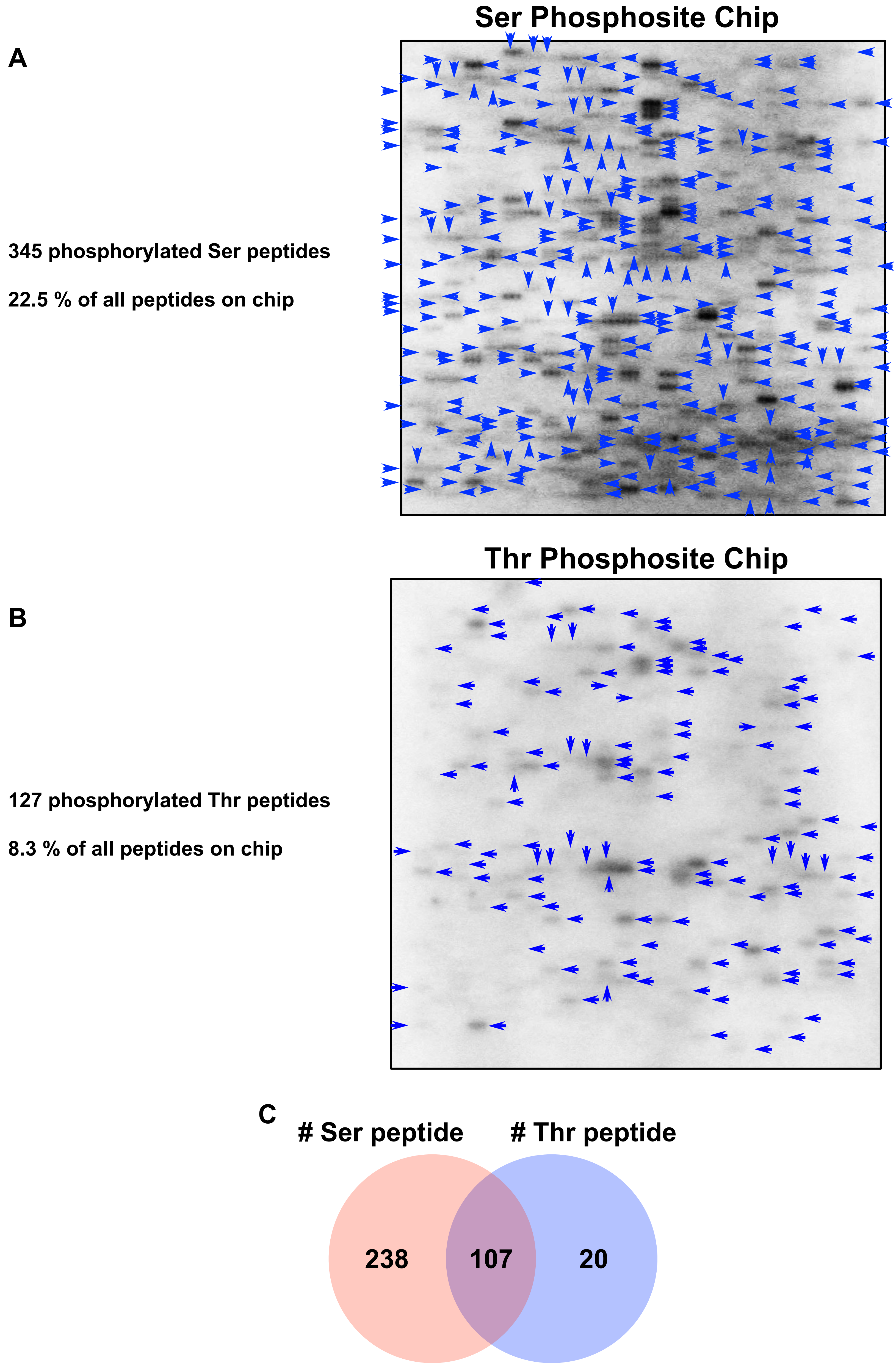


**S2 Fig. Position of all peptides phosphorylated by Adi3 on the Ser- and Thr-peptide microarrays**. (A) Position of phosphorylated peptides on the Ser-peptide microarray chip. (B) Position of phosphorylated peptides on the Thr-peptide microarray chip. (C) Number of phosphorylated peptides on Ser- or Thr- or both peptide microarrays.
