# Supplementary material for: Screening for potential nuclear substrates for the plant cell death suppressor kinase Adi3 using peptide microarrays
Source: PLoS One. 2020 Jun 2;15(6):e0234011. doi: 10.1371/journal.pone.0234011 (PMC7266335; doi:10.1371/journal.pone.0234011)
Supplement: S3 Fig — (PDF) [file pone.0234011.s003.pdf]

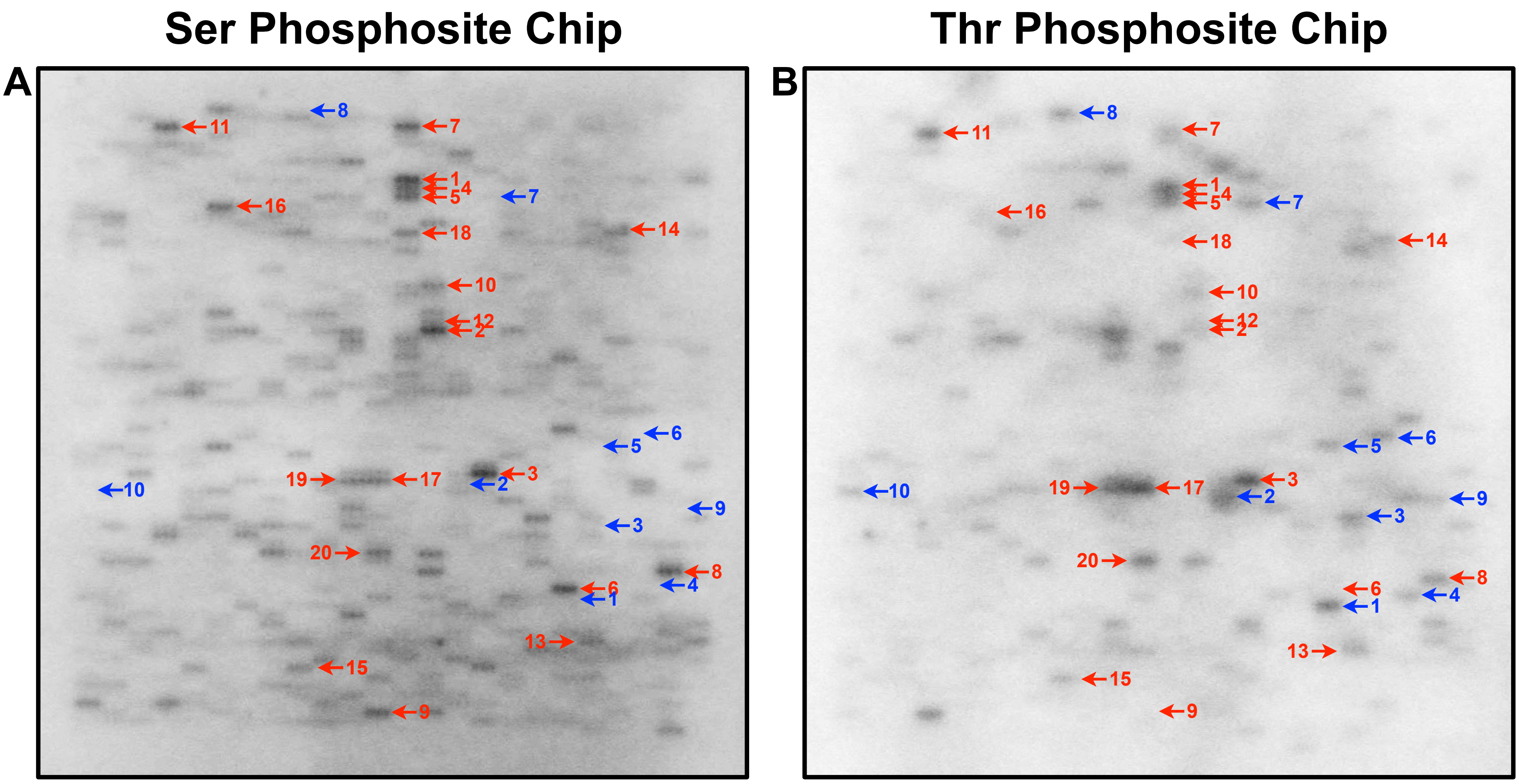

C

Peptide #	Sequence	Notes
1	LNWFEPS/THEPDYF	
2	AWFERPS/TVYFGHT	
3	AKLMDNS/TIPYLYV	
4	WLYGWGS/TQMSGDY	
5	IPELWVS/THHYTDN	
6	YMLMIIS/TGNLNAM	Thr peptide not phosphorylated
7	SAFEMPS/TAHYHFH	
8	FDLPDLS/TWVQSFD	
9	FTVFKLS/TSGSDHA	Thr peptide not phosphorylated
10	QWYWDKS/TFWWWHT	
11	LPVAVYS/TQVPEYW	
12	GEFNLHS/TGGERWF	
13	FDMIWFS/TGLEDVL	
14	DFFYGYS/TNMPNVV	
15	TVLIGYS/TPMGHFQ	
16	AFMFGQS/TSPPFYV	
17	FLALENS/TYYHYHG	
18	WVFYRIS/TNYDEPN	
19	WDKWQES/TWYWNGF	
20	PWDNYIS/TEPVQGY	

D

Peptide #	Sequence
1	FGIPTYTTYPNWP
2	WLTAEVTGEQWLY
3	KPRSSYTWKIYAS
4	AAFHWDTYAKKYY
5	EPGAWQTYFQLWP
6	WARNSVTYAWQHK
7	FKNHWMTYERFYL
8	WFDDKATFYWIYY
9	LKDFHITWLPMQL
10	YLQVHSTTYLTYN

S3 Fig. Mapping and comparison of Adi3 phosphorylated peptides on the Ser- and Thr-peptide microarray chips. (A) and (B), Red arrows and numbers show the position of the top 20 peptides phosphorylated by Adi3 on the Ser-peptide microarray chip and the corresponding location on the Thr-peptide microarray chip. Blue arrows and numbers show the position on both chips of the top 10 peptides phosphorylated by Adi3 only on the Thr-peptide microarray chip. (C) Sequence of the top 20 peptides phosphorylated on the Ser-peptide microarray chip and also phosphorylated on the Thr-peptide microarray chip. (D) Sequence of the 10 peptides phosphorylated only on the Thr chip.
