# Supplementary material for: Screening for potential nuclear substrates for the plant cell death suppressor kinase Adi3 using peptide microarrays
Source: PLoS One. 2020 Jun 2;15(6):e0234011. doi: 10.1371/journal.pone.0234011 (PMC7266335; doi:10.1371/journal.pone.0234011)
Supplement: S4 Fig — (PDF) [file pone.0234011.s004.pdf]

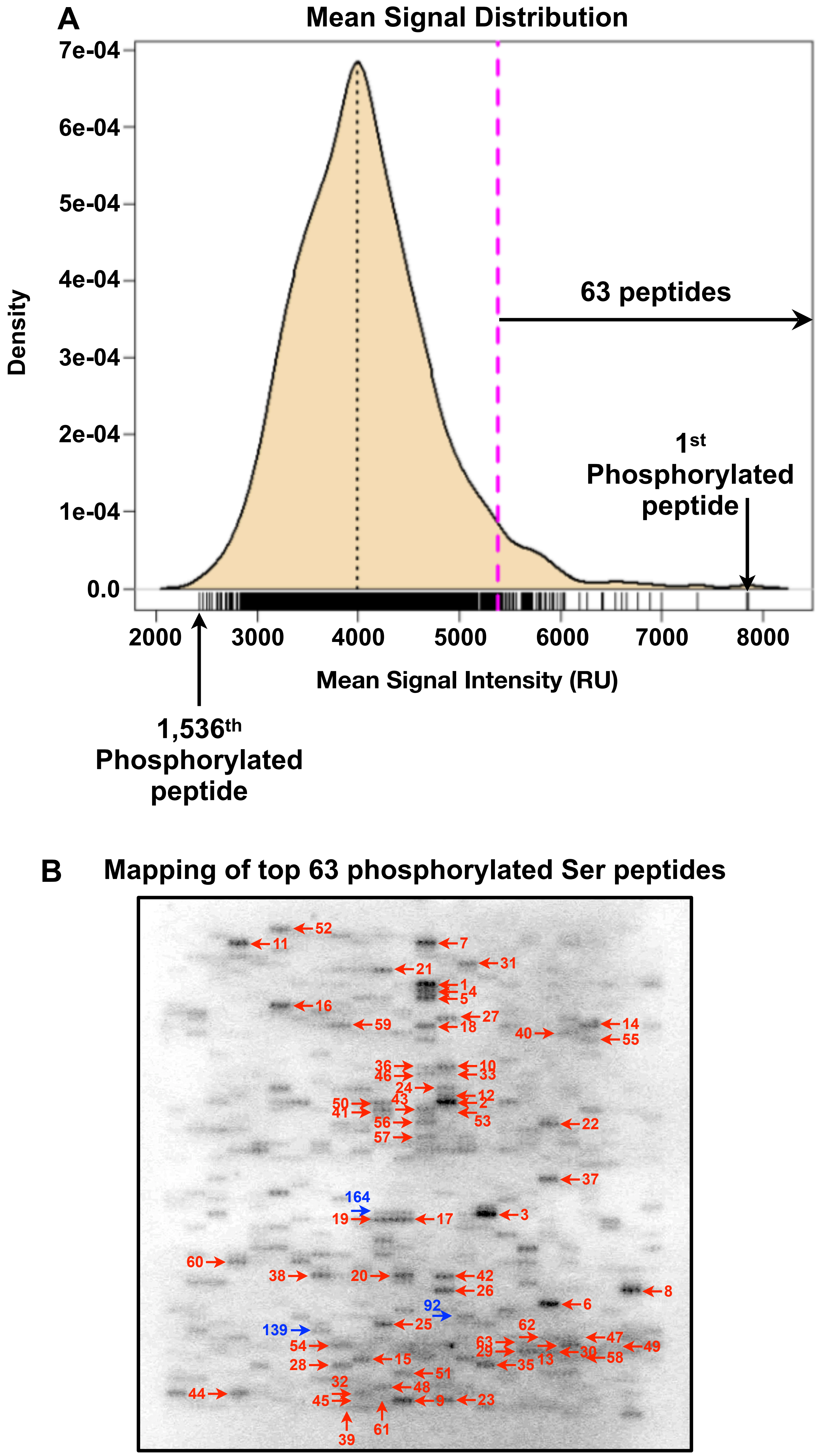

S4 Fig. Distribution of mean signal intensities for phosphorylated peptides and position of the top 63 Adi3 phosphorylated Ser peptides. (A) Probability density estimation for mean signal intensities (relative units, RU) of all 1,536 peptides on the Ser-peptide microarray chip. The dashed magenta line (5,384 RU) indicates 2 times the standard deviation from the maximum of distribution. The 63 peptides above this threshold were selected as queries. (B) Mapping of the top 63 phosphorylated peptides on the phosphorimage of one subarray of the Ser-peptide microarray chip (red arrows). The 92nd, 139th and 164th phosphorylated peptides were additionally mapped (blue arrows).
