# Supplementary material for: Screening for potential nuclear substrates for the plant cell death suppressor kinase Adi3 using peptide microarrays
Source: PLoS One. 2020 Jun 2;15(6):e0234011. doi: 10.1371/journal.pone.0234011 (PMC7266335; doi:10.1371/journal.pone.0234011)
Supplement: S5 Fig — (PDF) [file pone.0234011.s005.pdf]

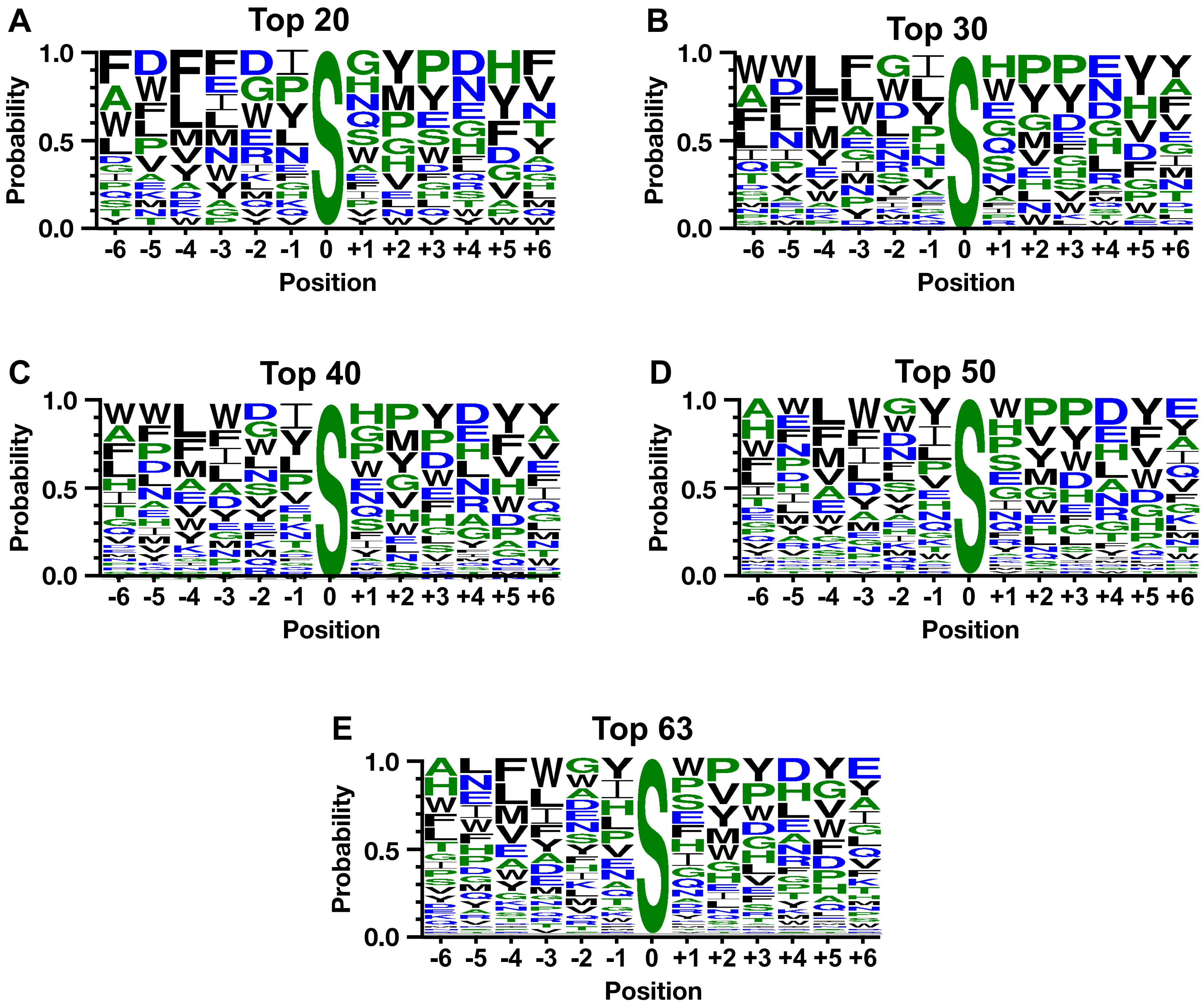

S5 Fig. Sequence logos based on different numbers of Adi3-phosphorylated peptides. Sequence logos for the top (A) 20, (B) 30, (C) 40, (D) 50, (E) 63 Ser peptides phosphorylated by Adi3.
