# Supplementary material for: Screening for potential nuclear substrates for the plant cell death suppressor kinase Adi3 using peptide microarrays
Source: PLoS One. 2020 Jun 2;15(6):e0234011. doi: 10.1371/journal.pone.0234011 (PMC7266335; doi:10.1371/journal.pone.0234011)
Supplement: S6 Fig — (PDF) [file pone.0234011.s006.pdf]

A

MEEVGCSSGKEKISNGSTRMDFDIDDLFEVDDDDWDEDEDEDEDDSHDMVLIKELGESFLKNFCKKASTGFFEKYGS
ISHQINSYNDFINYGIQRVFDSVGEIHVEPGYDPSKRGDGDWKHASVKFGKVTLERPKFWAGEKFSVGGGKEYLDLL
PRHARLQNMTYSARIMVETHVQVYTKKLVRSDKFKTGVDRFVDKEWEVEGKRDVLIGRIPVMVNSELCWMNGVDKLD
CEFDHGGYFIAKGAEKTFIAQEQLCLKRLWVSNNPTWMVGYRPGEKRKRVYIKLTETLKLEHIKGGEKALSVYILAE
MPIWVLFFALGVSSDREVVNLIDVDIEDTTIVNILVASIHEADKNCEDFRKGKKALAYVDRLIKNCKFPPQESVEEC
INAYLFPNLSGFKQKARFLGYMVKCLLHSFIGRRKVDNRDDFRNKRLELAGELLERELRAHIKHAERRMVKAMQRDL
YGDRQVQPIEHYLDASIITNGLSRAFSTGHWCHPYKRMERVSGVVATLRRTNPLQMTADMRKSRQQVTYTGKVGDAR
YPHPSHWGKLCFLSTPDGENCGLVKNLASMGLVSTIILKPFLETLFRCGMQKLVDDCSTSLHGKQKVLLDGEWVGVC
EDSALFVSKLRRKRRRNEVPHQVEVKRDELQGEVRIFSDPGRILRPLLVVSNLKKIKALKGGDYGFQSLLDNGIIEL
IGPEEEEDCRTAWGVEYILKADKENPPAKYTHCELDMSFLLGLSCGIIPFANHDHARRVLYQSEKHSQQAIGFSTVN
PNNRVDTNTHQLYYPQRPLFRTMLADSLGKPKCAQYQKGMLPRPEYFNGQCAIVAVNVHLGYNQEDSLVMNRASLER
GMFRSEHVRSYKAEVDNKEAMAKKLKIEDSVNFGKTQSKIGRVDSLDDDGFPFIGANLQSGDIIIGKFSESGADHSV
KLKHTERGMVQKVLLSANDEGKNFAVVSLRQVRSPCLGDKFSSMHGQKGVLGFLESQENFPFTIQGIVPDIVINPHA
FPSRQTPGQLLEAALGKGIALGGGQKYATPFSTLSVDAIMEQLQGRGFTRWGNERVYNGRTGEMVHSLIFMGPTFYQ
RLIHMAEDKVKFRNTGPVHPLTRQPVADRKRFGGIKFGEMERDCLIAHGAAANLHERLFTLSDSSQMHICGKCKNMA
NVIQRTVQGGKVRGPFCRFCESVEDIVKVNVPYGAKLLCQELFSMGISLKFDTEIC

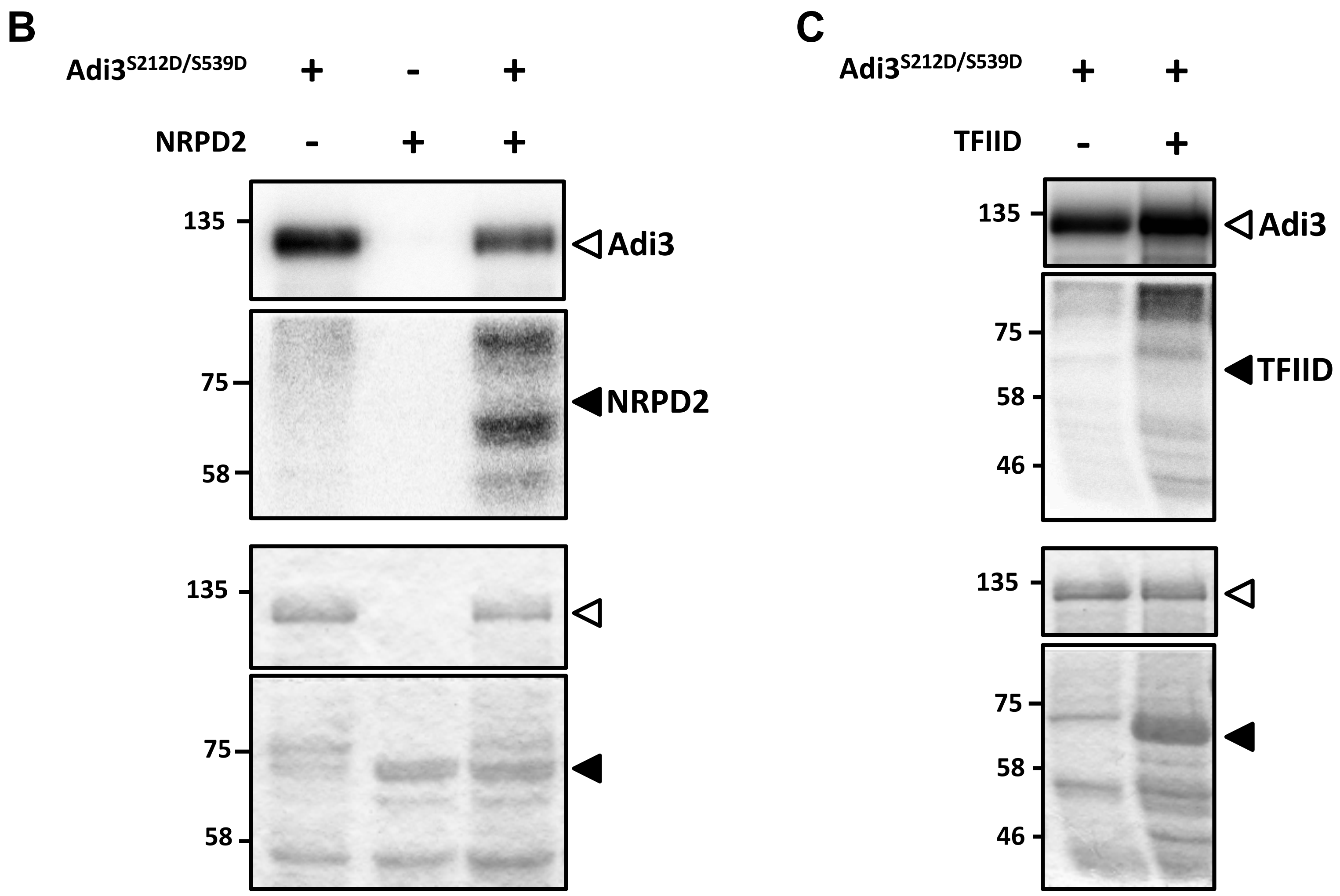

S6 Fig. Adi3 does not phosphorylate NRPD2 or TFIID. (A) Amino acid sequence of NRPD2. The underlined bold sequence domain was expressed and purified to test Adi3-mediated phosphorylation. The potentially phosphorylated Ser residues are in red. In B and C, Adi3 *in vitro* kinase activity toward (C) NRPD2 and (D) TFIID. Three μg of NRPD2 or TFIID was incubated with 1 μCi of [γ-^{32}P]ATP in the presence of 1μg of Adi3$^{S212D/S595D}$. Top and bottom pair of panels show the phosphorimage and Coomassie stained gel, respectively. Experiments were repeated three times with similar results.
