# Supplementary material for: Screening for potential nuclear substrates for the plant cell death suppressor kinase Adi3 using peptide microarrays
Source: PLoS One. 2020 Jun 2;15(6):e0234011. doi: 10.1371/journal.pone.0234011 (PMC7266335; doi:10.1371/journal.pone.0234011)
Supplement: S7 Fig — (PDF) [file pone.0234011.s007.pdf]

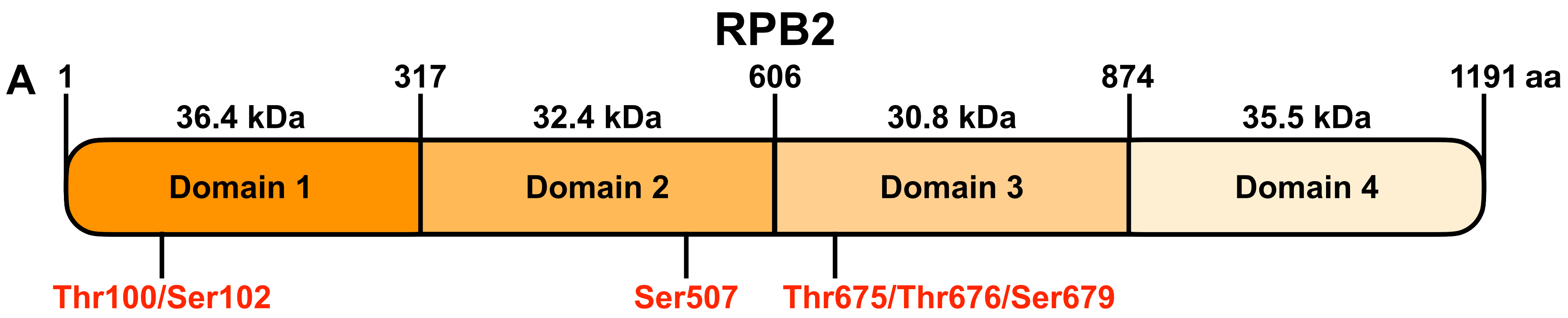

B

MDMEDEYEPQYNVDDDEEEITQEDAWAVISAYFEEKGLVRQQLDSFDEFIQNTMQEIVDESADI
EIRPESQHNPGHQSDFAETIYKINFGQIYLSKPMMTESDGETATLFPKAARLRNLTYSAPLYVD
VTKRVIKKGHDGEEVTETQDFTKVFIGKVPIMLRSSYCTLYQNSEKDLTELGECPLDQGGYFII
NGSEKVLIAQEKMSTNHVYVFKKRQPNKYAFVAEVRSMADTQNRPPSTMFVRMLSRTSAKGGSS
GQYIRATLPYIRTEIPIIIVFRALGFVADKDILEHICYDFNDTQMMELLRPSLEEAFVIQNQQV
ALDYIGKRGATVGVTREKRIKYAKEILQKEMLPHVGVGEYCETKKAYYFGYIIHRLLLCALGRR
AEDDRDHYGNKRLDLAGPLLGGLFRMLFRKLTRDVRGYVQKCVDNGKDVNLQFAIKAKTITSGL
KYSLATGNWGQANAAGTRAGVSQVLNRLTYASTLSHLRRLNSPIGREGKLAKPRQLHNSQWGMM
CPAETPEGQACGLVKNLALMVYITVGSAAYPILEFLEEWGTENFEEISPAVIPQATKIFVNGTW
VGIHRDPDMLVRTLRRLRRRVDVNTEVGVVRDIRLKELRIYTDYGRCSRPLFIVEKQRLMIKKK
DIQTLQQRESPDEGGWHDLVAKGYIEYIDTEEEETTMISMTINDLVQARLNPGDAYSDTYTHCE
IHPSLILGVCASIIPFPDHNQSPRNTYQSAMGKQAMGIYVTNYQFRMDTLAYVLYYPQKPLVTT
RAMEHLHFRQLPAGINAIVAISCYSGYNQEDSVIMNQSSIDRGFFRSLFFRSYRDEEKKMGTLV
KEDFGRPDRASTMGMRHGSYDKLDDDGLAPPGTRVSGEDVIIGKTTPISQDDAQGQASRYTRKD
HSTSLRHSETGMVDQVLLTTNADGLRFVKVRVRSVRIPQIGDKFSSRHGQKGTVGMTYTQEDMP
WTVEGITPDIIVNPHAIPSRMTIGQLIECIMGKVAAHMGKEGDATPFTDVTVDNISKALHKCGY
QMRGFETMYNGHTGRRLSAMIFLGPTYYQRLKHMVDDKIHSRGRGPVQILTRQPAEGRSRDGGL
RFGEMERDCMIAHGAAHFLKERLFDQSDAYRVHVCERCGLIAIANLKKNSFECRGCKNKTDIVQ
VHIPYACKLLFQELMAMAIAPRMLTKDVKLAKDQKKKGA

S7 Fig. Protein domains of RNA polymerase II, second largest subunit (RPB2). (A) Representation of the RPB2 protein delineated into the four domains used for protein expression. Amino acid positions and molecular weight of each domain are given. Positions of potential Ser phosphorylation sites based on peptide alignment are shown in red lettering. (B) Amino acid sequence of RPB2 with the color highlighted regions matching the domains shown in A. Underlined sequences correspond to the portions of peptides 48, 62, and 139, in domains 1, 2 and 3, respectively, that matched RPB2 in the BLAST search. The potentially phosphorylated Ser or Thr are in red or blue lettering, respectively.
