# Supplementary material for: Screening for potential nuclear substrates for the plant cell death suppressor kinase Adi3 using peptide microarrays
Source: PLoS One. 2020 Jun 2;15(6):e0234011. doi: 10.1371/journal.pone.0234011 (PMC7266335; doi:10.1371/journal.pone.0234011)
Supplement: S8 Fig — (PDF) [file pone.0234011.s008.pdf]

A

Peptide Sequence	Peptide #	BLAST Results			
		Query	2	LNDNDS	7
YLNDNDSTVLAEW	164			LN+NDS	
		Pti5	11	LNENDS	16

B MVPTPQSDLPLNENDSQEMVLYEVLNEANALNIPYLPQRNQ
LLPRNNILRPLQCIGKKYRGVRRRPWGKYAAEIRDSARHGA
RVWLGTFETAEEAALAYDRAAFRMRGAKALLNFPSEIVNAS
VSVDKLSLCSNSYTTNNNSDSSLNEVSSGTNDVFESRC

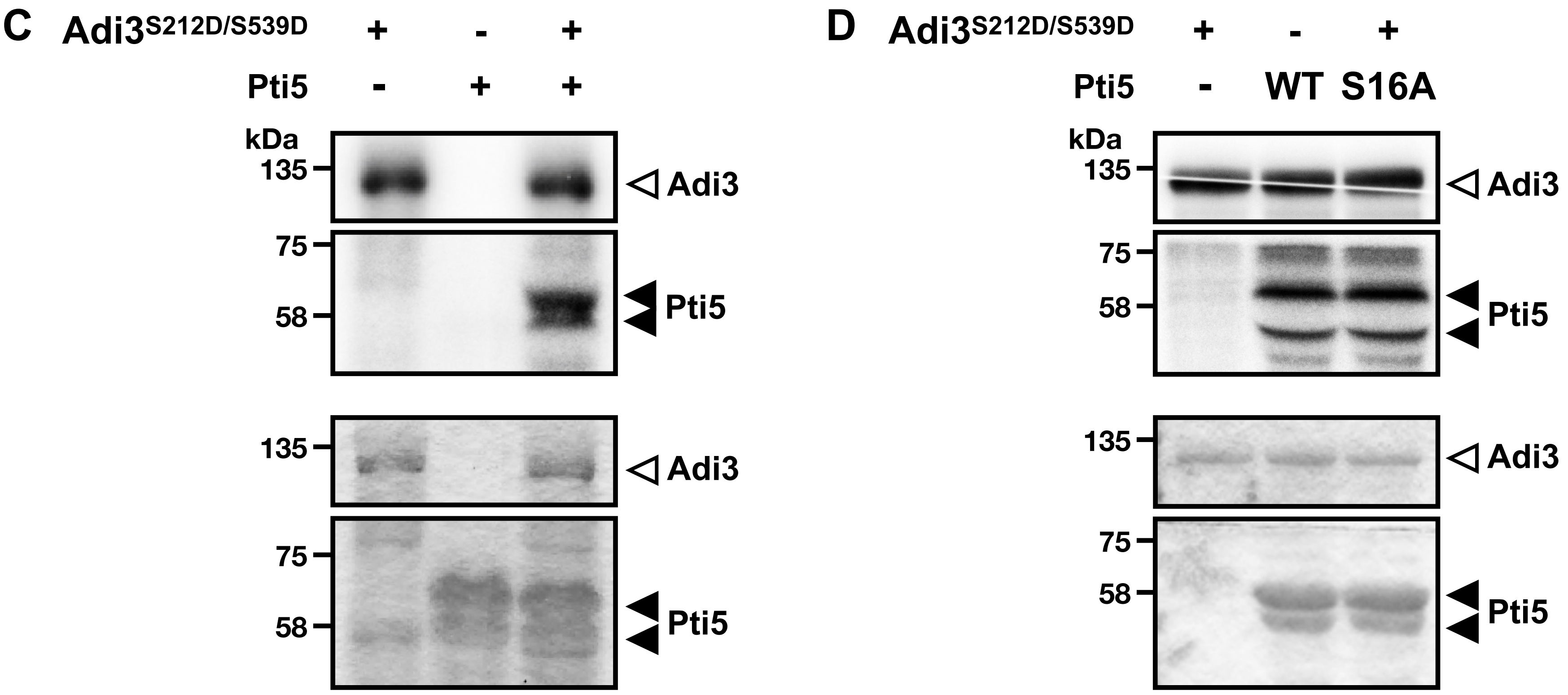

S8 Fig. Identification and analysis of Pti5 as a potential Adi3 substrate. (A) BLAST results from the identification of Pti5 as a potential Adi3 substrate. Pti5 was identified by BLAST using the 164th peptide as a query. In the peptide sequence column, the Ser residue highlighted in red indicates possible phosphorylation site. Peptide # refers to the ranking of the indicated peptide used for BLAST. In the BLAST results column, numbers represent amino acid positions in the peptide or Pti5 protein. (B) Pti5 amino acid sequence. Underlined sequences correspond to the portions of peptides 164 that matched Pti5 in the BLAST search. The potentially phosphorylated Ser is in red lettering. In C and D, Adi3 *in vitro* kinase activity toward (C) Pti5^{WT} and (D) Pti5^{S16A}. Three μg of Pti5^{WT} or Pti5^{S16A} was incubated with 1 μCi of [γ-^{32}P]ATP in the presence of 1μg of Adi3$^{S212D/S595D}$. Top and bottom pair of panels show the phosphorimage and Coomassie stained gel, respectively. Experiments were repeated three times with similar results.
