# Supplementary material for: Screening for potential nuclear substrates for the plant cell death suppressor kinase Adi3 using peptide microarrays
Source: PLoS One. 2020 Jun 2;15(6):e0234011. doi: 10.1371/journal.pone.0234011 (PMC7266335; doi:10.1371/journal.pone.0234011)
Supplement: S9 Fig — (PDF) [file pone.0234011.s009.pdf]

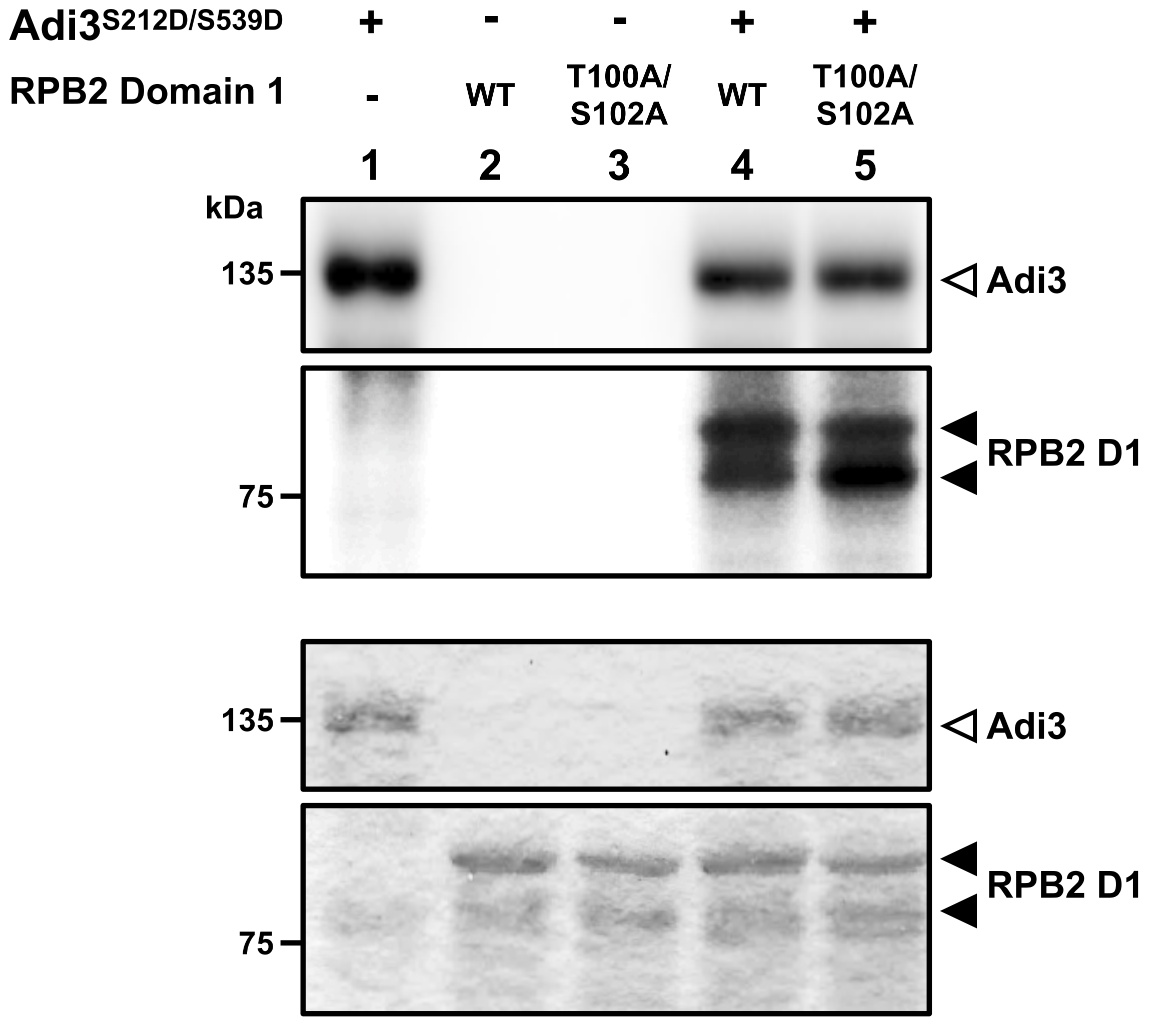

S9 Fig. Adi3 does not phosphorylate RPB2 domain 1 (D1) at T100 or S102. *in vitro* kinase activity of Adi3 toward RPB2 D1. Three μg of the indicated RPB2 D1 point mutants were incubated with 1 μCi of [γ-^{32}P]ATP in the presence of 1μg of Adi3$^{S212D/S595D}$. Top and bottom pair of panels show the phosphorimage and Coomassie stained gel, respectively. Experiments were repeated three times with similar results.
