# Supplementary material for: Screening for potential nuclear substrates for the plant cell death suppressor kinase Adi3 using peptide microarrays
Source: PLoS One. 2020 Jun 2;15(6):e0234011. doi: 10.1371/journal.pone.0234011 (PMC7266335; doi:10.1371/journal.pone.0234011)
Supplement: S1 Table — (PDF) [file pone.0234011.s010.pdf]

S1 Table. Primers used in this study.

Gene ID	Gene name	Primer Name	Purpose	Forward primer (5’ to 3’)	Reverse primer (5’ to 3’)
Solyc01g090240	Adi3	Adi3-S212D	Mutagenesis*	GTTGTGAGATCTATGgacATTGTCAACAGTTGC	GCAACTGTTGACAATgtcCATAGATCTCACAAC
		Adi3-S539D		CCTACTTCAGCACGGTCAATGgatTTTGTTGGGACTCATGAATATTTG	CAAATATTCATGAGTCCCAACAAAatcCATTGACCGTGCTGAAGTAGG
Solyc12g099970	Gal83	Gal83-S26T	Mutagenesis*	CGGTCAGGTATCGGGAAGAAGAactAATGTTGAATCTGG	CCAGATTCAACATTagtTCTTCTTCCCGATACCTGACCG
Solyc02g078260	RPB2	RPB2	ORF Amplification	CTAAAACTAAGCAGACAGGATCTGGGTTCCG	CGAGCCTCTTGTACATGAAAAGCAGGAG
		RPB2-D1	Cloning into pMAL-c2x	GCGGGATCC(*Bam*HI)ATGGATATGGAGGATGAATATG	GGCGTCGAC(*Sal*I)TCAGTTTTGAATAACAAAAGCTTC
		RPB2-D3		GTGGGATCC(*Bam*HI)CGTGATATCCGTTTGAAAGAAC	GGCGTCGAC(*Sal*I)TCAAATGATAACATCCTC ACCA
		RPB2-D4		TAAGGATCC(*Bam*HI)GGGAAGACCACTCCCATTTCT	TATGTCGAC(*Sal*I)CGAGCCTCTTGTACATGAAAAG
		RPB2-D1-T100A/S102A	Mutagenesis*	CAATGATGgcaGAGgcaGATGGT	ACCATCtgcCTCtgcCATCATTG
		RPB2-D3-T675A		GAGGAAGAAgcaACGATGATTAGCATGACTATAAATG	TTCAGTGTCAATATACTCGATGTATCCCTTAGCCACG
		RPB2-D3-T676A		GAGGAAGAAACAgcgATGATTAGCATGACTATAAATG	
		RPB2-D3-S679A		GAGGAAGAAACAACGATGATTgccATGACTATAAATG	
		RPB2-D3-T675A/T676A		GAGGAAGAAgcagcgATGATTAGCATGACTATAAATG	
		RPB2-D3-T675A/S679A		GAGGAAGAAgcaACGATGATTgccATGACTATAAATG	
		RPB2-D3-T676A/S679A		GAGGAAGAAACAgcgATGATTgccATGACTATAAATG	
		RPB2-D3-T675A/T676A/S679A		GAGGAAGAAgcagcgATGATTgccATGACTATAAATG	
Solyc02g077370	Pti5	Pti5	ORF Amplification	GCTATGGTTCCAACTCCTCAAAGTGATTTACCTC	CGTGTCCACACATTATTCGCTTAGAGTGC
		Pti5-WT	Cloning into pMAL-c2x	GGCCGAATTC(*Eco*RI)ATGGTTCCAACTCCTCAAAGTGATTTACC	TGTCTGCAG(*Pst*I)CAAGAAATTCTCCATGCACAGCTCTG
		Pti5-S16A	Mutagenesis*	GAGAATGACgcaCAAGAGATGG	CCATCTCTTGtgcGTCATTCTC

*Lower case letters indicate the substituted nucleotides for mutants
