# Supplementary material for: Screening for potential nuclear substrates for the plant cell death suppressor kinase Adi3 using peptide microarrays
Source: PLoS One. 2020 Jun 2;15(6):e0234011. doi: 10.1371/journal.pone.0234011 (PMC7266335; doi:10.1371/journal.pone.0234011)
Supplement: S2 Table — (PDF) [file pone.0234011.s011.pdf]

S2 Table. Sequence of the top 63 phosphorylated Ser peptides

#	Sequence	Name	Mean Signal Intensity
1	LNWFEPSHEPDYF	Random-Ser-0766	7853
2	AWFERPSVYFGHT	Random-Ser-0687	7837
3	AKLMDNSIPYLYV	Random-Ser-0508	7355
4	WLYGWGSQMSGDY	Random-Ser-0870	7345
5	IPELWVSHHYTDN	Random-Ser-0964	6999
6	YMLMIISGNLNAM	Random-Ser-0004	6884
7	SAFEMPSAHYHFH	Random-Ser-0213	6758
8	FDLPDLSWVQSFD	Random-Ser-1522	6654
9	FTVFKLSSGSDHA	Random-Ser-1386	6608
10	QWYWDKSFWWWHT	Random-Ser-0218	6545
11	LPVAVYSQVPEYW	Random-Ser-0271	6423
12	GEFNLHSGGERWF	Random-Ser-0593	6410
13	FDMIWFSGLEDVL	Random-Ser-0572	6265
14	DFFYGYSNMPNVV	Random-Ser-1324	6190
15	TVLIGYSPMGHFQ	Random-Ser-0814	6047
16	AFMFGQSSPPFYV	Random-Ser-1125	6030
17	FLALENSYYHYHG	Random-Ser-0633	6025
18	WVFYRISNYDEPN	Random-Ser-1340	6002
19	WDKWQESWYWNGF	Random-Ser-0630	5968
20	PWDNYISEPVQGY	Random-Ser-1385	5936
21	TISFWYSEPYAVM	Random-Ser-0628	5919
22	IIMWLLSHNYEEE	Random-Ser-0942	5892
23	NYYRSASSWDLNG	Random-Ser-1347	5852
24	HWLDFVSQYGEDA	Random-Ser-0499	5840
25	WLEWSTSHPFMVI	Random-Ser-0349	5813
26	AYWANLSWGPLPI	Random-Ser-1533	5804
27	WHPLYRSWEDRYA	Random-Ser-1250	5801
28	MNLLNHSELHHYY	Random-Ser-0904	5799
29	QFMPLTSYVKNWY	Random-Ser-0705	5797
30	LNLGGISRIVGYE	Random-Ser-0662	5789
31	EENYSYSMPLAWA	Random-Ser-0502	5787
32	HPWVVDSFVFDQE	Random-Ser-1284	5757
33	GMAINKSHHWIQW	Random-Ser-0312	5756
34	HWIAKISPSELWP	Random-Ser-0986	5724
35	RPVWDVSPAYYAV	Random-Ser-0885	5712
36	VSETMYSKWHAFQ	Random-Ser-0214	5708
37	LHGIVESGGRRPI	Random-Ser-0003	5701
38	TFAWFPSNSDRFL	Random-Ser-1369	5684
39	IAKDYISIMWDMQ	Random-Ser-1472	5671
40	VQLDQWSPMIHVY	Random-Ser-1414	5664
41	HNAWHVSEVWDDK	Random-Ser-0816	5648
42	YEVLGNSWLHTGG	Random-Ser-1346	5633
43	HREYAQSSYLTVK	Random-Ser-0777	5620
44	AEVWFYSPQPDTS	Random-Ser-1307	5609
45	PMFQAGSSQWLWE	Random-Ser-1378	5566
46	KRMFNWSWPGAYI	Random-Ser-0308	5565
47	AIGIHHSDEDFGE	Random-Ser-0478	5545
48	DYMMTESRWYELE	Random-Ser-1194	5544
49	HHTDIYSLVPHFQ	Random-Ser-0670	5538
50	EGSWWQSIPPDWK	Random-Ser-0722	5533
51	YNYYEASFIVHWT	Random-Ser-1010	5527
52	KQFTIESEYAPGI	Random-Ser-0091	5523
53	TGREEMSFVHFQH	Random-Ser-0781	5503
54	GLGIPISAWYMGA	Random-Ser-0623	5496
55	SEVETPSIHRWIL	Random-Ser-1512	5471
56	PITLGHSWMVKDY	Random-Ser-0965	5449
57	HLEWKHSSVTYGP	Random-Ser-1153	5429
58	VHFVSVSIIGDAR	Random-Ser-0760	5418
59	FQNRYTSTPYPLE	Random-Ser-1371	5417
60	LGVWAHSPTSLPL	Random-Ser-1213	5408
61	ALWQMLSDYTKVG	Random-Ser-1382	5396
62	SIMLHNSFWGHSE	Random-Ser-0474	5392
63	WNFAAASQDLPPS	Random-Ser-0611	5390
92	DIVWEKSVEYGPQ	Random-Ser-0224	5214
139	WETAMISSNWYTS	Random-Ser-0431	4988
164	YLNDNDSTVLAEW	Random-Ser-0536	4890
