# Supplementary material for: Screening for potential nuclear substrates for the plant cell death suppressor kinase Adi3 using peptide microarrays
Source: PLoS One. 2020 Jun 2;15(6):e0234011. doi: 10.1371/journal.pone.0234011 (PMC7266335; doi:10.1371/journal.pone.0234011)
Supplement: S3 Table — (PDF) [file pone.0234011.s012.pdf]

# S3 Table. Final 11 potential Adi3 phosphorylation candidates.

| | Annotation | Peptide BLAST resutls | Peptide # | Peptide Sequence | NCBI ID Protein | NCBI ID Gene | Uniprot ID | Gene ID | *A. thaliana* Gene ID |
|---|---|---|---|---|---|---|---|---|---|
| 1 | **RNA polymerase II 2nd largest subunit RPB2** | Query 3 MMTES 7<br>MMTES<br>Sbjct 118 MMTES 122<br>5/5(100%) | 48 | DYMMTESRWYELE | NP_001233889.1 | 544278 | Q42877 | Solyc02g078260.2.1 | AT4G21710 |
| | | Query 4 LHNSFWG 10<br>LHNS WG<br>Sbjct 523 LHNSHWG 529<br>6/7(86%) | 62 | SIMLHNSFWGHSE | | | | | |
| | | Query 2 ETAMIS 7<br>ET MIS<br>Sbjct 674 ETTMIS 679<br>5/6(83%)<br>Query 2 ETAMI 6<br>ET M+<br>Sbjct 905 ETGMV 909<br>3/5(60%) | 139 | WETAMISSNWYTS | | | | | |
| 2 | **RNA polymerase IV 2nd largest subunit NRPD2** | Query 4 LWVSHHYT 11<br>LWVS HYT<br>Sbjct 264 LWVSNHYT 271<br>7/8(88%) | 5 | IPELWVSHHYTDN | XP_004245914.1 | 101249196 | K4CMW1 | Solyc08g075940.2 | AT3G18090 |
| | | Query 3 EWKHSSVTYG 12<br>EWKH SV +G<br>Sbjct 128 EWKHASVKFG 137<br>7/10(70%) | 57 | HLEWKHSSVTYGP | | | | | |
| 3 | **Histone demethylase** | Query 4 MDNSIP 9<br>MDNSIP<br>Sbjct 819 MDNSIP 824<br>6/6(100%) | 3 | AKLMDNSIPYLYV | XP_004236784.1 | 101246913 | K4BPF4 | Solyc04g009990.2 | AT1G08620 |
| | | Query 3 VWEKS 7<br>VWEKS<br>Sbjct 188 VWEKS 192<br>5/5(100%) | 92 | DIVWEKSVEYGPQ | | | | | |
| 4 | **Transcription elongation factor SPT5** | Query 9 MSGDY 13<br>MSGDY<br>Sbjct 354 MSGDY 358<br>5/5(100%) | 4 | WLYGWGSQMSGDY | XP_004237729.1 | 101260813 | K4BT61 | Solyc04g064700.2 | AT2G34210 |
| 5 | **RNA polymerase I-specific transcription initiation factor RRN3** | Query 7 SGNLNA 12<br>SGNLNA<br>Sbjct 278 SGNLNA 283<br>6/6(100%) | 6 | YMLMIISGNLNAM | XP_004232589.1 | 101266087 | K4BAA4 | Solyc02g082340.2 | AT2G34750 |
| 6 | **Zinc finger CCCH domain-containing protein 19 NERD** | Query 3 FE-MPSA 8<br>FE MPSA<br>Sbjct 1230 FEKMPSA 1236<br>6/7(86%) | 7 | SAFEMPSAHYHFH | XP_010313216.1 | M_010314914. | K4D933 | Solyc11g062220.1 | AT2G16485 |
| 7 | **Zinc finger CCCH domain protein Oxidative Stress 2 (OX2)** | Query 4 PDLSWVQS 11<br>PDLSWVQS<br>Sbjct 638 PDLSWVQS 645<br>8/8(100%) | 8 | FDLPDLSWVQSFD | XP_004249271.1 | 101258208 | K4D2R6 | Solyc10g080260.1 | AT2G41900 |
| 8 | **Transcription initiation factor TFIID subunit 11** | Query 5 KLSSGSD 11<br>KLSS SD<br>Sbjct 92 KLSSSSD 98<br>6/7(86%) | 9 | FTVFKLSSGSDHA | XP_004239004.1 | 101252686 | K4BZ67 | Solyc05g018120.1 | AT4G20280 |
| 9 | **26S proteasome regulatory subunit 4 homolog A** | Query 6 HSGGER 11<br>HSGGER<br>Sbjct 302 HSGGER 307<br>6/6(100%) | 12 | GEFNLHSGGERWF | XP_004242383.1 | 101267617 | K4CAL7 | Solyc06g083620.2 | AT4G29040 |
| 10 | **Apoptotic chromatin condensation inducer** | Query 6 VSEVWD 11<br>VSEVWD<br>Sbjct 251 VSEVWD 256<br>6/6(100%)<br>Query 5 HVS--EVWDDK 13<br>H+S E +DK<br>Sbjct 539 HISKKE--NDK 547<br>5/11(45%) | 41 | HNAWHVSEVWDDK | NP_001334103.1 | 101245055 | K4BTQ4 | Solyc04g072670.2 | AT4G39680 |
| 11 | **Pto-interacting 5 Pti5** | Query 2 LNDNDS 7<br>LN+NDS<br>Sbjct 11 LNENDS 16<br>5/5(100%) | 164 | YLNDNDSTVLAEW | NP_001233987.1 | 544042 | O04681 | Solyc02g077370.1 | AT3G23240 |
