# Supplementary material for: Screening for potential nuclear substrates for the plant cell death suppressor kinase Adi3 using peptide microarrays
Source: PLoS One. 2020 Jun 2;15(6):e0234011. doi: 10.1371/journal.pone.0234011 (PMC7266335; doi:10.1371/journal.pone.0234011)
Supplement: S1 Raw images — (PDF) [file pone.0234011.s015.pdf]

Fig. 2A or B

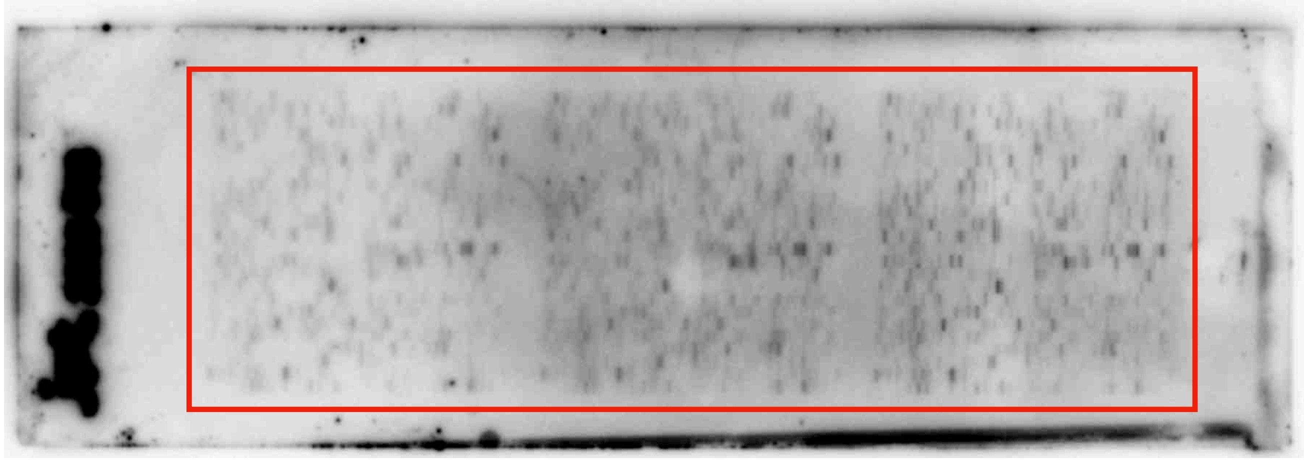

Fig. 2C

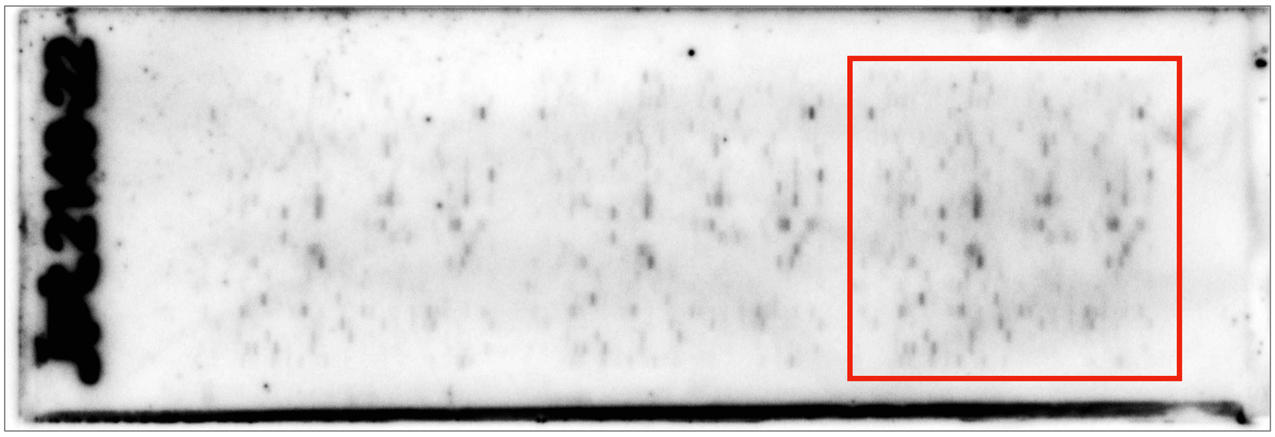

Fig. 2D

Adi3$^{S212D/S539D}$ | Adi3$^{S212D/S539D}$ + Gal83 | Adi3$^{S212D/S539D}$ + Gal83^{S26T}

Phosphorimage

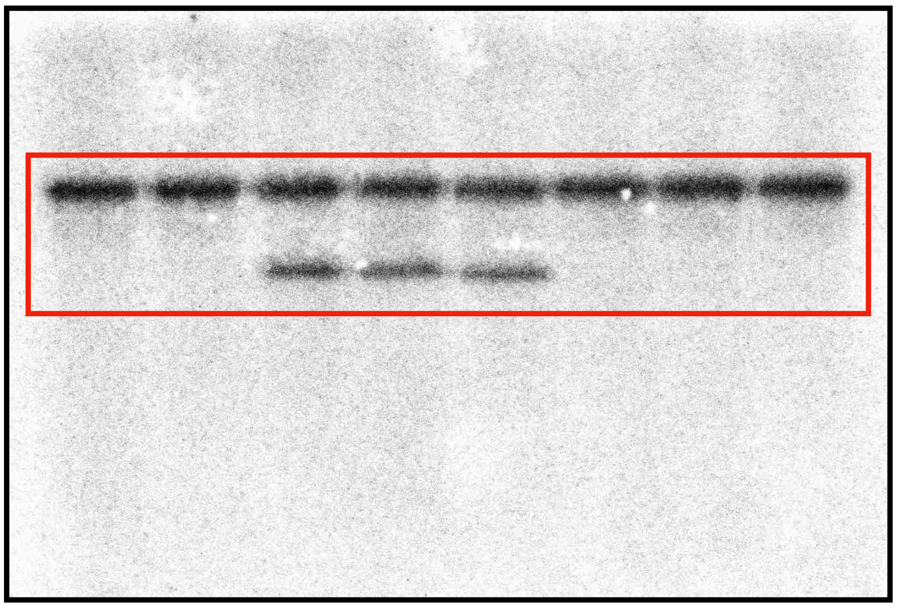

Marker | Adi3$^{S212D/S539D}$ | Adi3$^{S212D/S539D}$ + Gal83 | Adi3$^{S212D/S539D}$ + Gal83^{S26T}

Stained gel

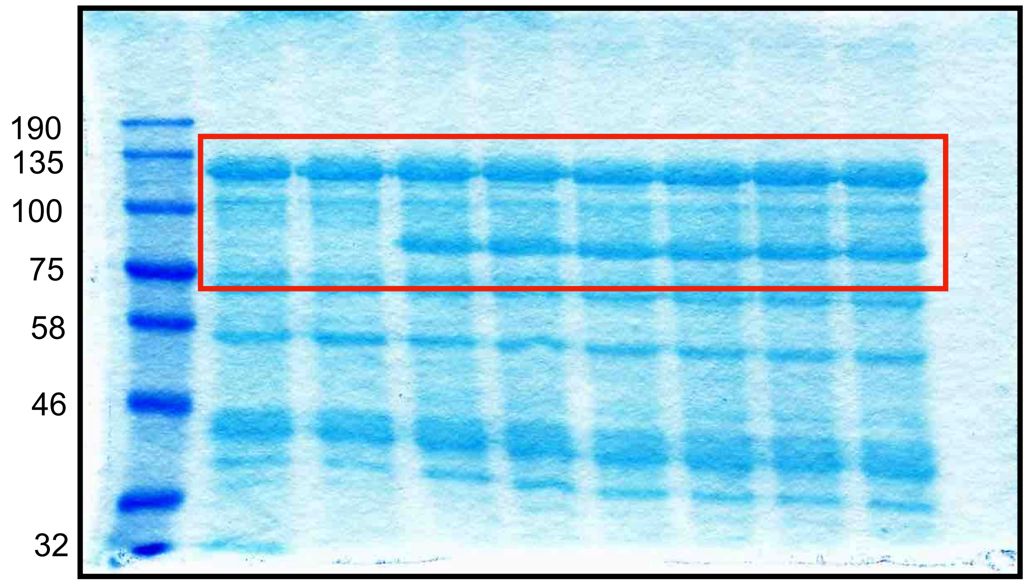

Fig. 5B

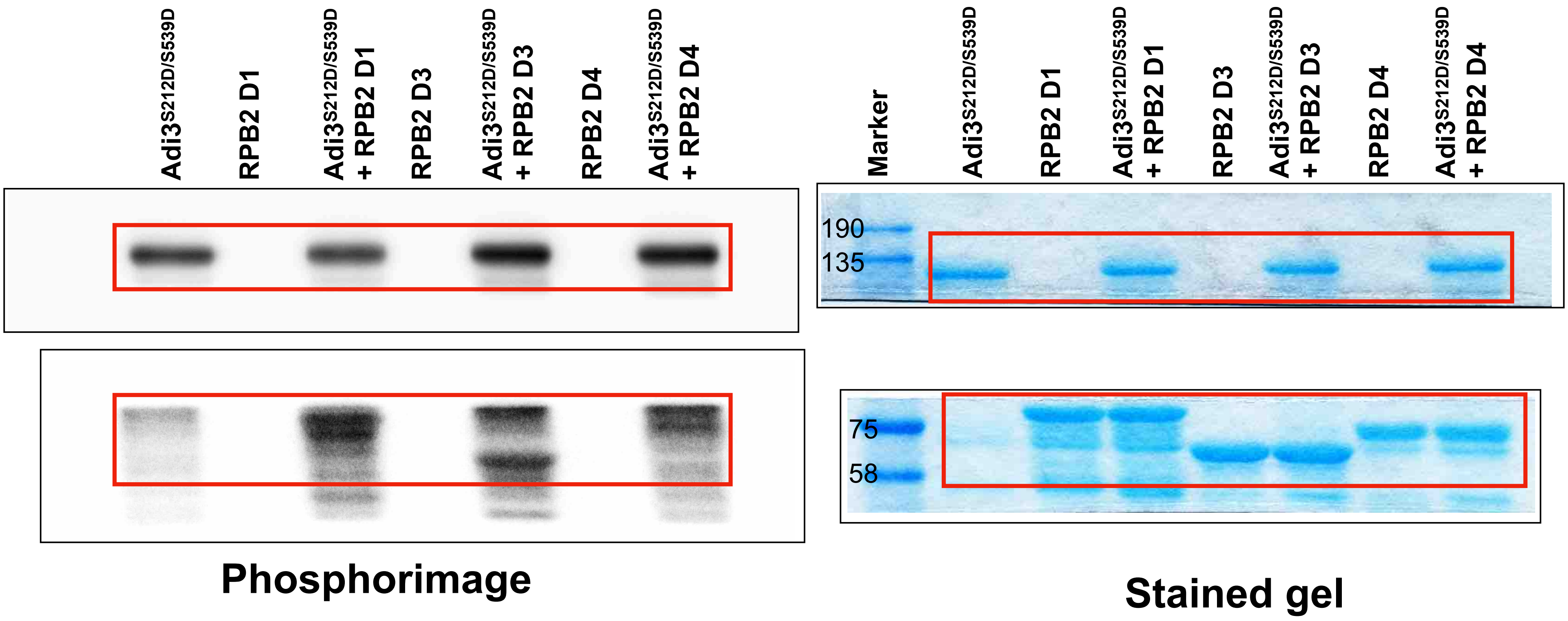
Adi3$^{S212D/S539D}$
RPB2 D1
Adi3$^{S212D/S539D}$ + RPB2 D1
RPB2 D3
Adi3$^{S212D/S539D}$ + RPB2 D3
RPB2 D4
Adi3$^{S212D/S539D}$ + RPB2 D4
Phosphorimage
Marker
Adi3$^{S212D/S539D}$
RPB2 D1
Adi3$^{S212D/S539D}$ + RPB2 D1
RPB2 D3
Adi3$^{S212D/S539D}$ + RPB2 D3
RPB2 D4
Adi3$^{S212D/S539D}$ + RPB2 D4
190
135
75
58
Stained gel

Fig. 5C

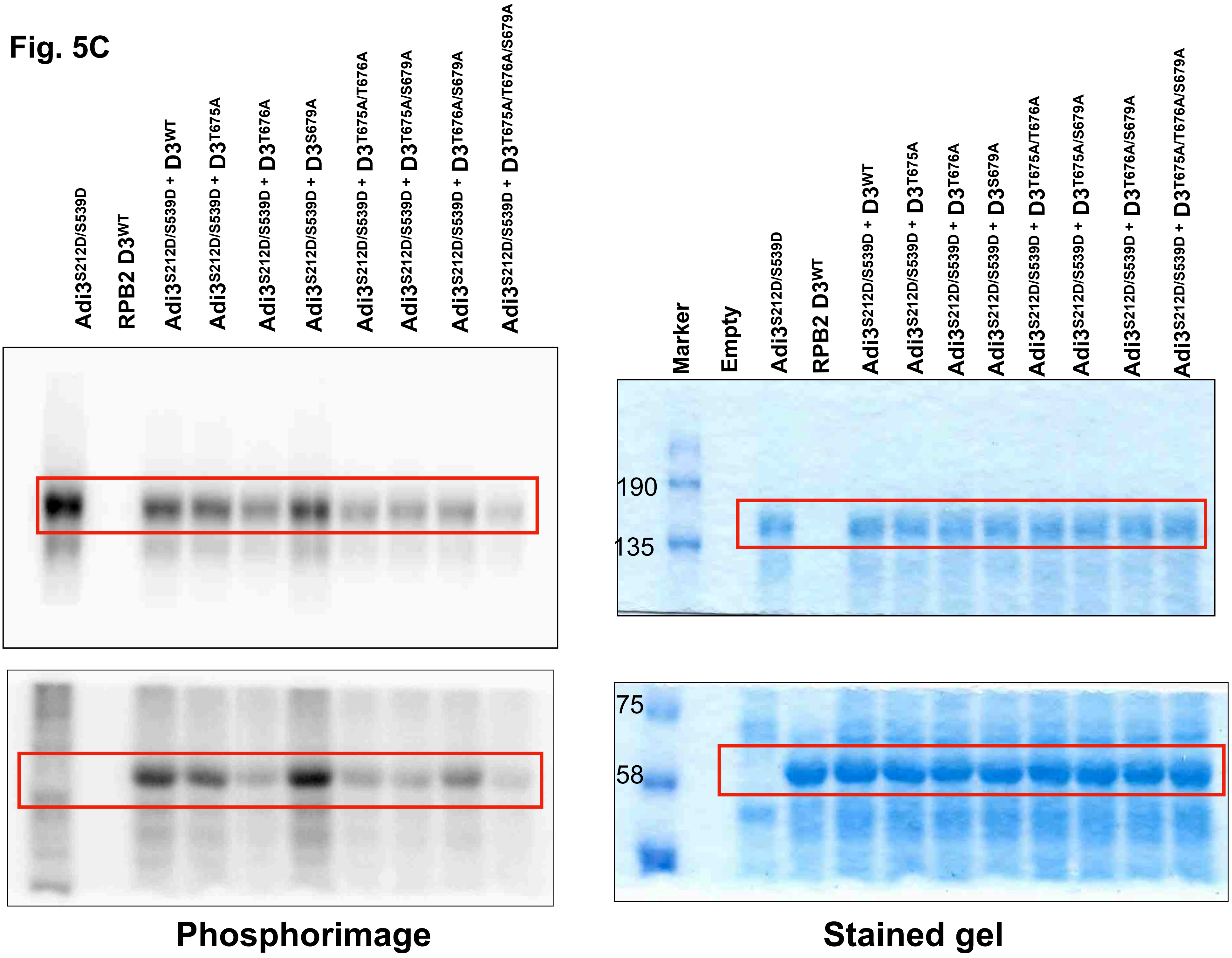
Adi3$^{S212D/S539D}$
RPB2 D3^{WT}
Adi3$^{S212D/S539D}$ + D3^{WT}
Adi3$^{S212D/S539D}$ + D3^{T675A}
Adi3$^{S212D/S539D}$ + D3^{T676A}
Adi3$^{S212D/S539D}$ + D3^{S679A}
Adi3$^{S212D/S539D}$ + D3$^{T675A/T676A}$
Adi3$^{S212D/S539D}$ + D3$^{T675A/S679A}$
Adi3$^{S212D/S539D}$ + D3$^{T676A/S679A}$
Adi3$^{S212D/S539D}$ + D3$^{T675A/T676A/S679A}$
Phosphorimage
Marker
Empty
Adi3$^{S212D/S539D}$
RPB2 D3^{WT}
Adi3$^{S212D/S539D}$ + D3^{WT}
Adi3$^{S212D/S539D}$ + D3^{T675A}
Adi3$^{S212D/S539D}$ + D3^{T676A}
Adi3$^{S212D/S539D}$ + D3^{S679A}
Adi3$^{S212D/S539D}$ + D3$^{T675A/T676A}$
Adi3$^{S212D/S539D}$ + D3$^{T675A/S679A}$
Adi3$^{S212D/S539D}$ + D3$^{T676A/S679A}$
Adi3$^{S212D/S539D}$ + D3$^{T675A/T676A/S679A}$
190
135
75
58
Stained gel

Fig. S1A

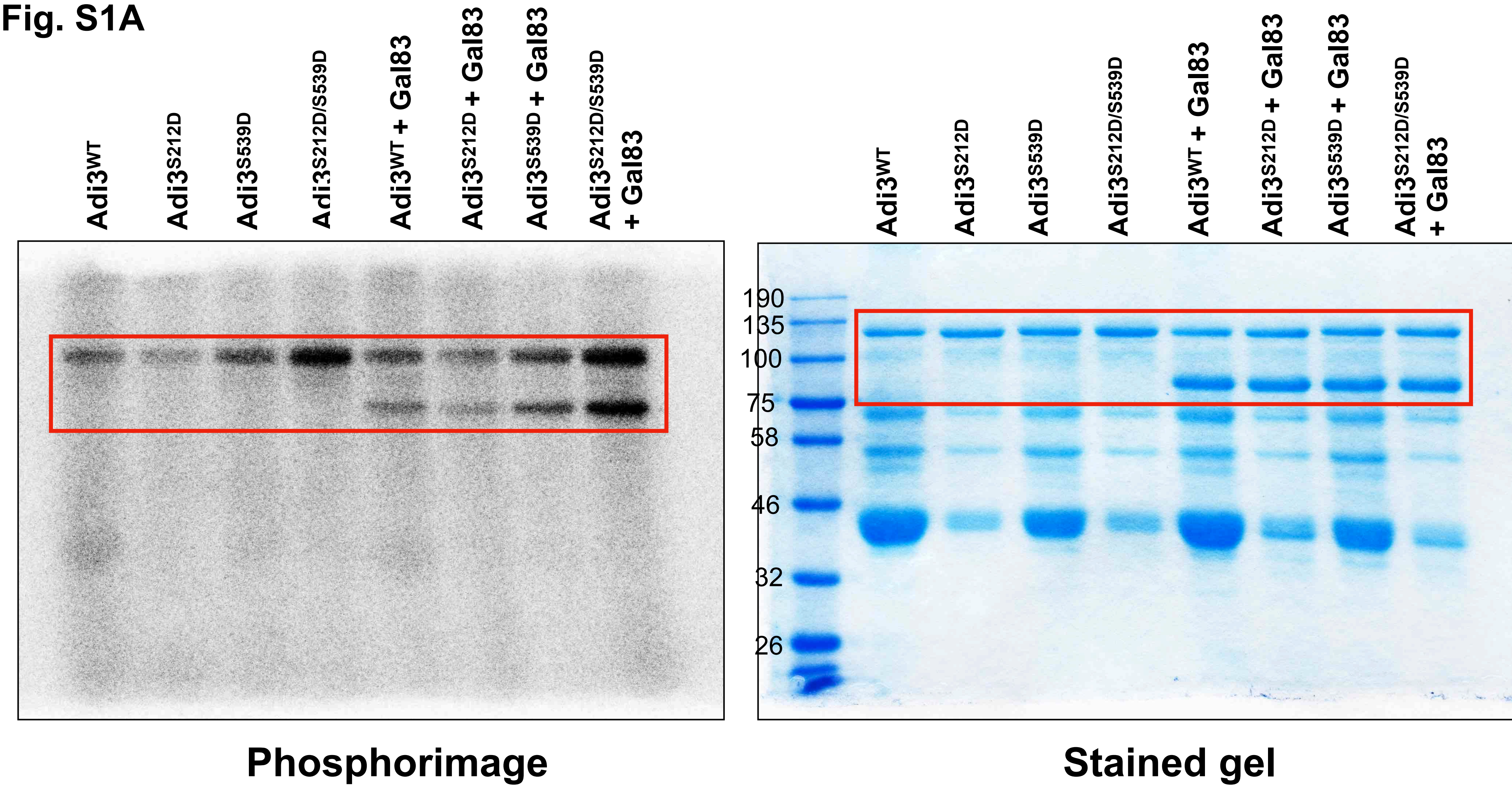

Fig. S1B

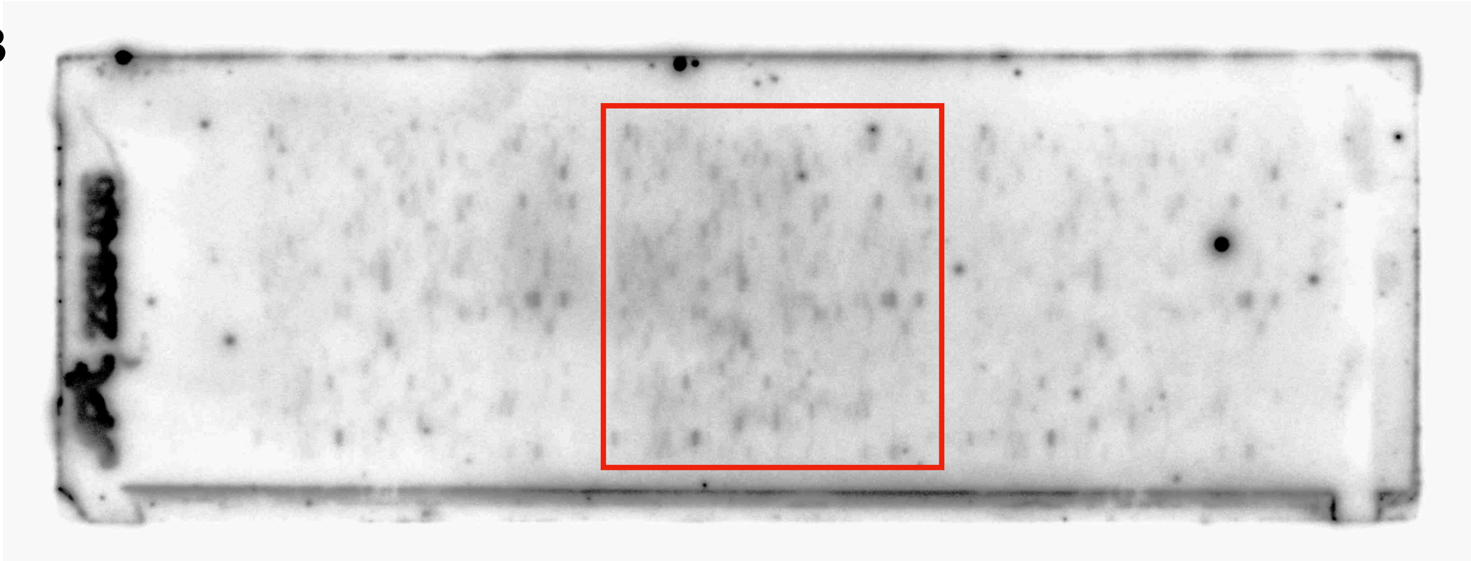

Fig. S1C

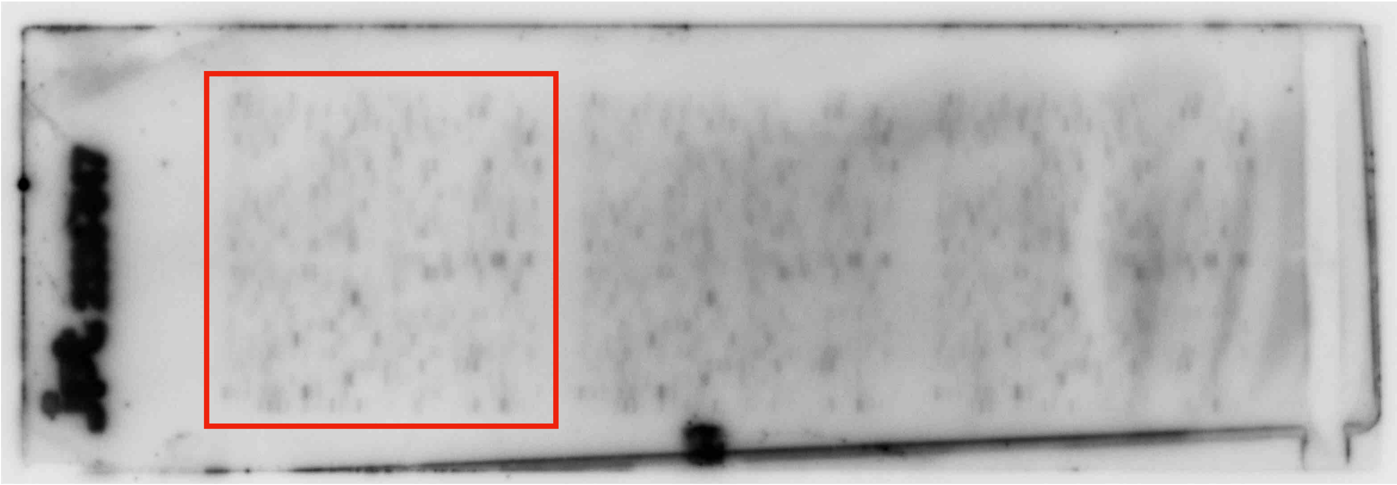

Fig. S1D - The image in Fig. 2C was used.

Fig. S2A - The image in Fig. 2A was used.

Fig. S2B - The image in Fig. 2C was used.

Fig. S3A - The image in Fig. 2A was used.

Fig. S3B - The image in Fig. 2C was used.

Fig. S4B - The image in Fig. 2A was used.

Fig. S6B

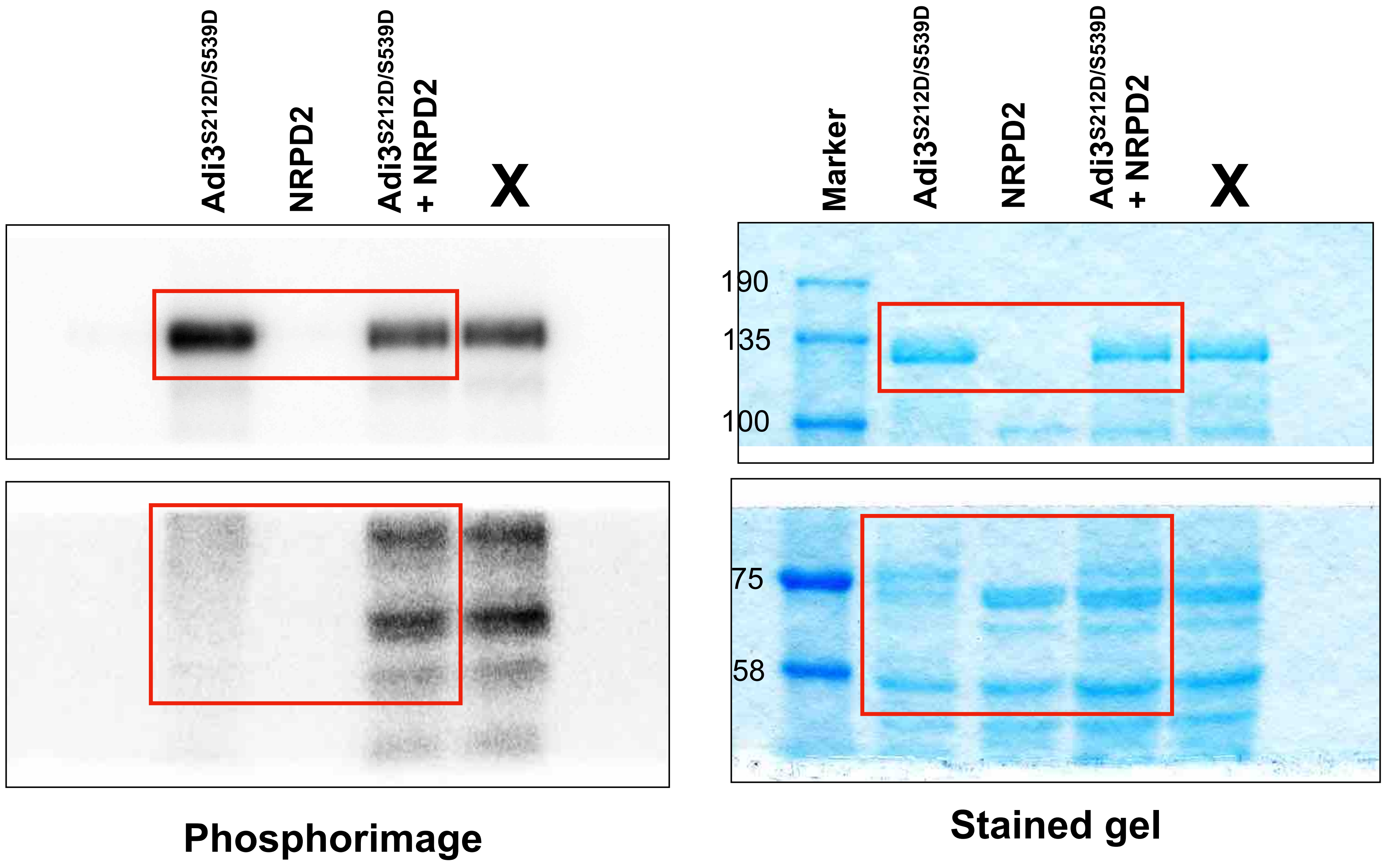

Fig. S6C

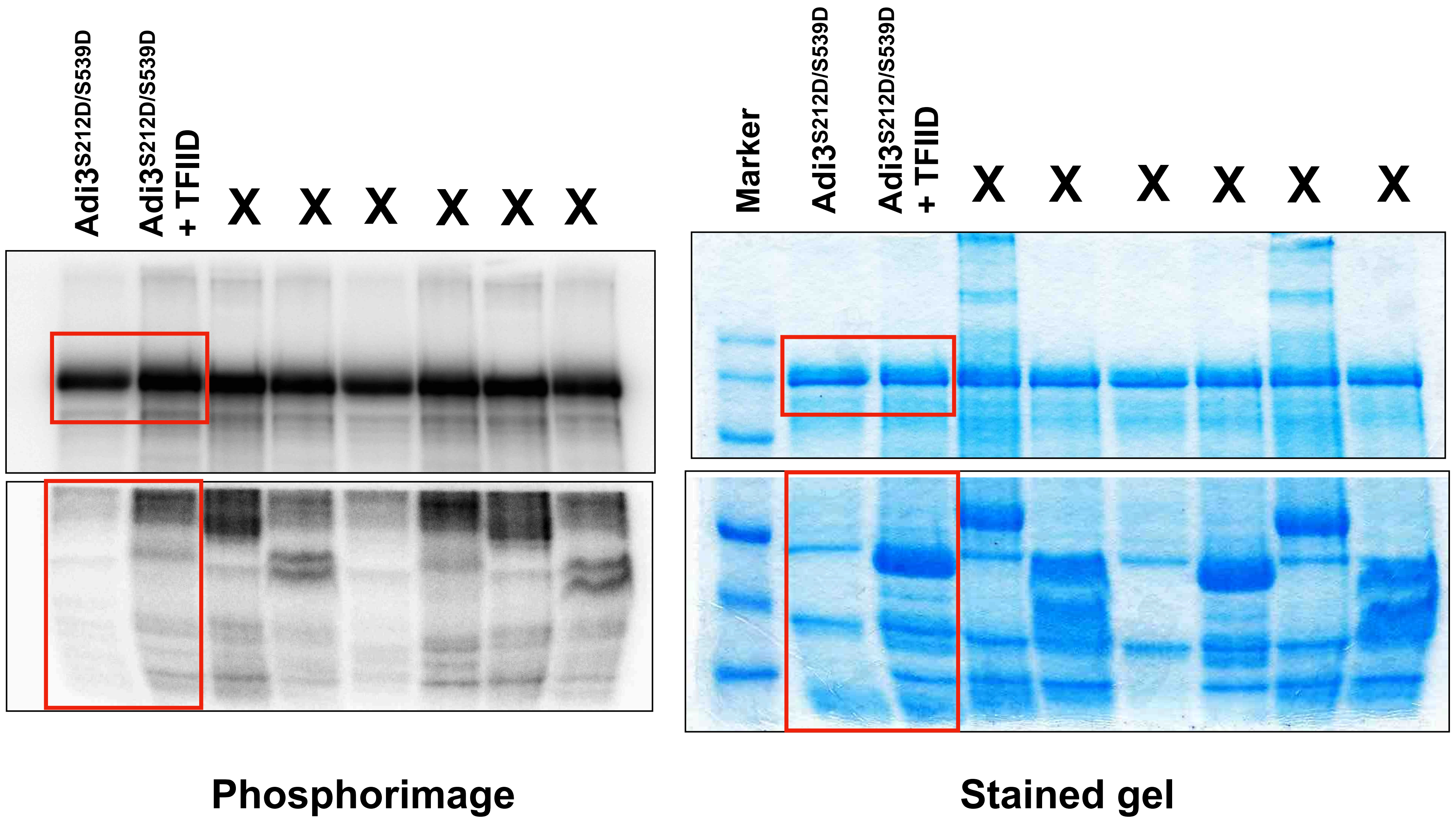

Fig. S8C

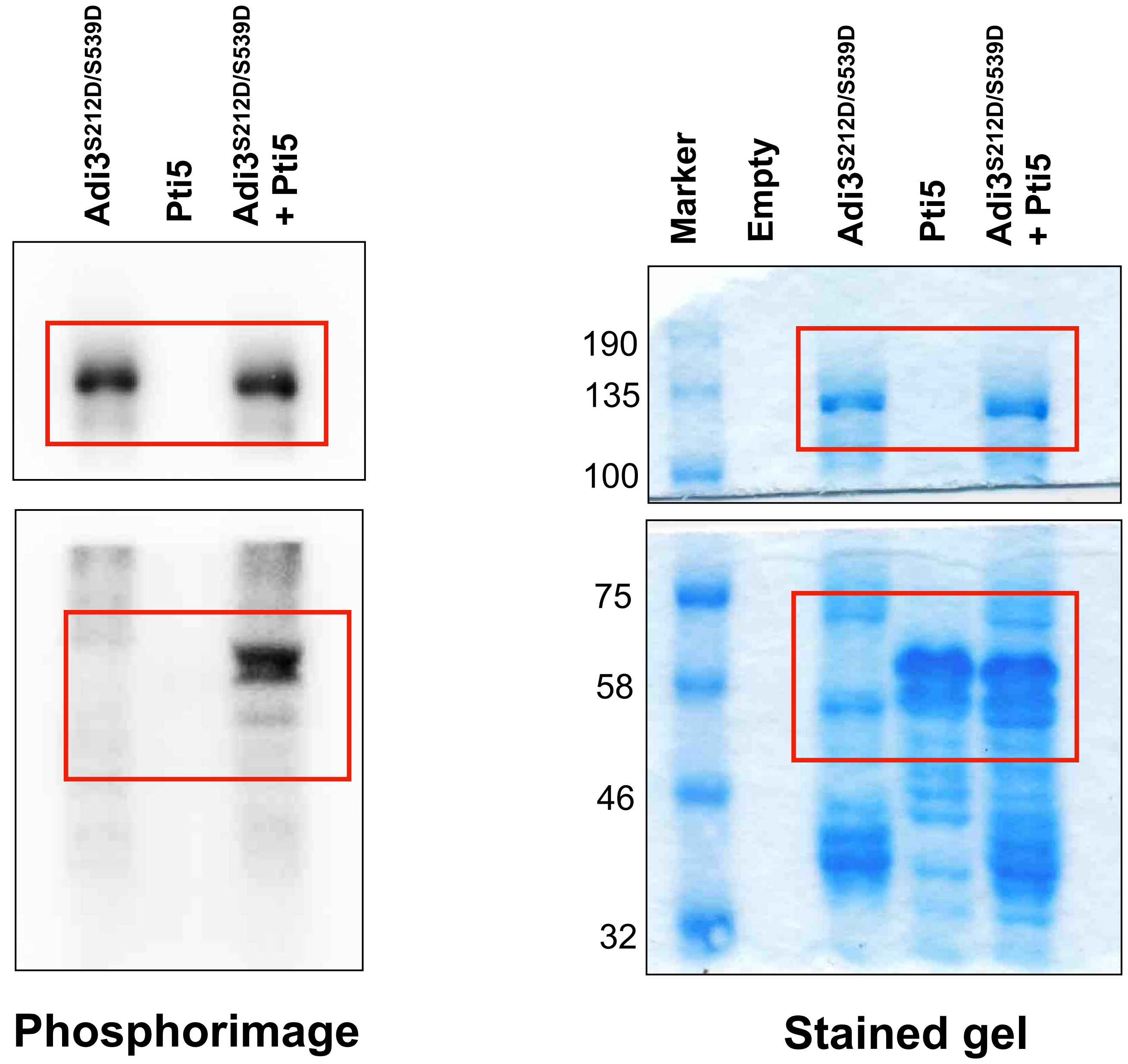

Fig. S8D

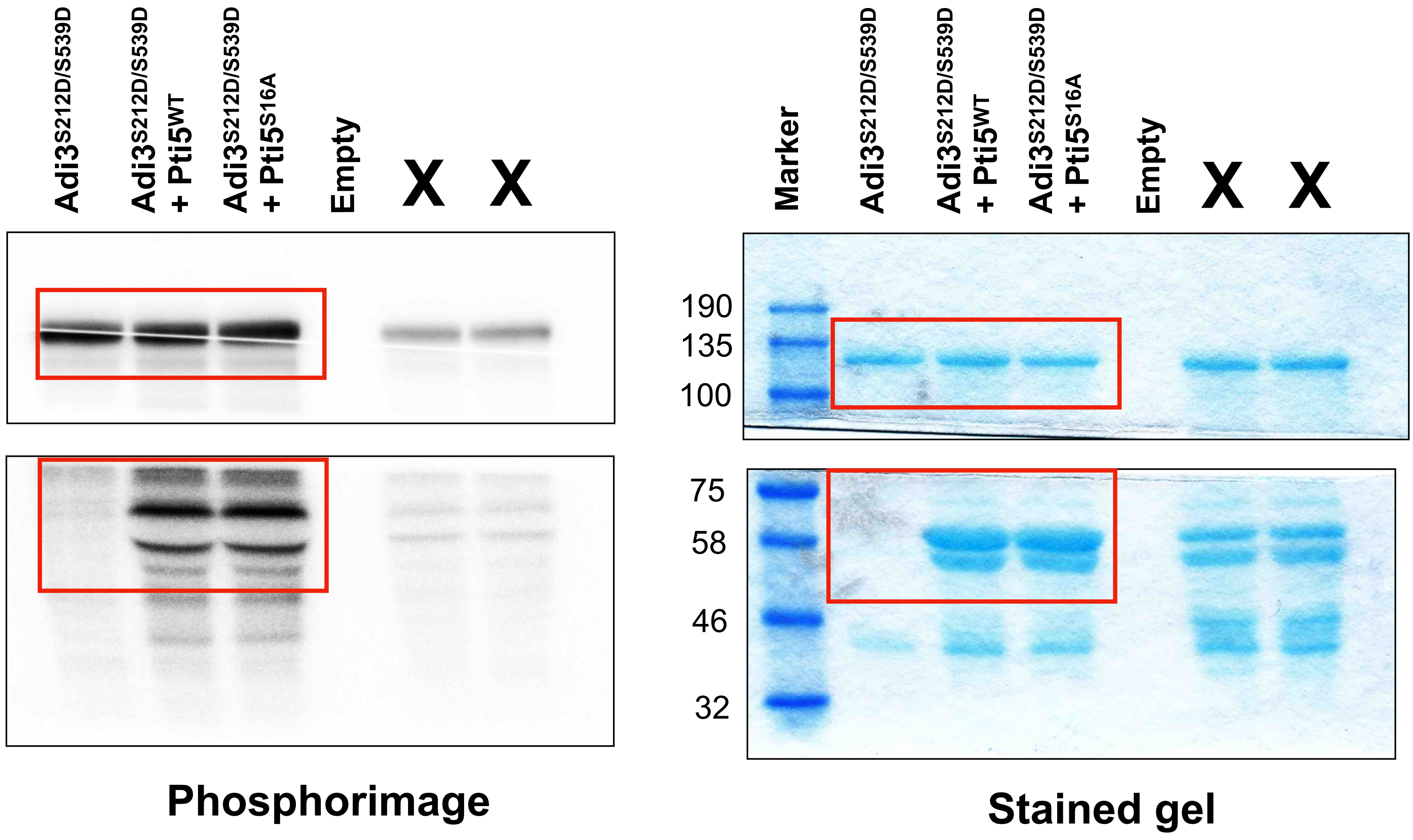

Fig. S9

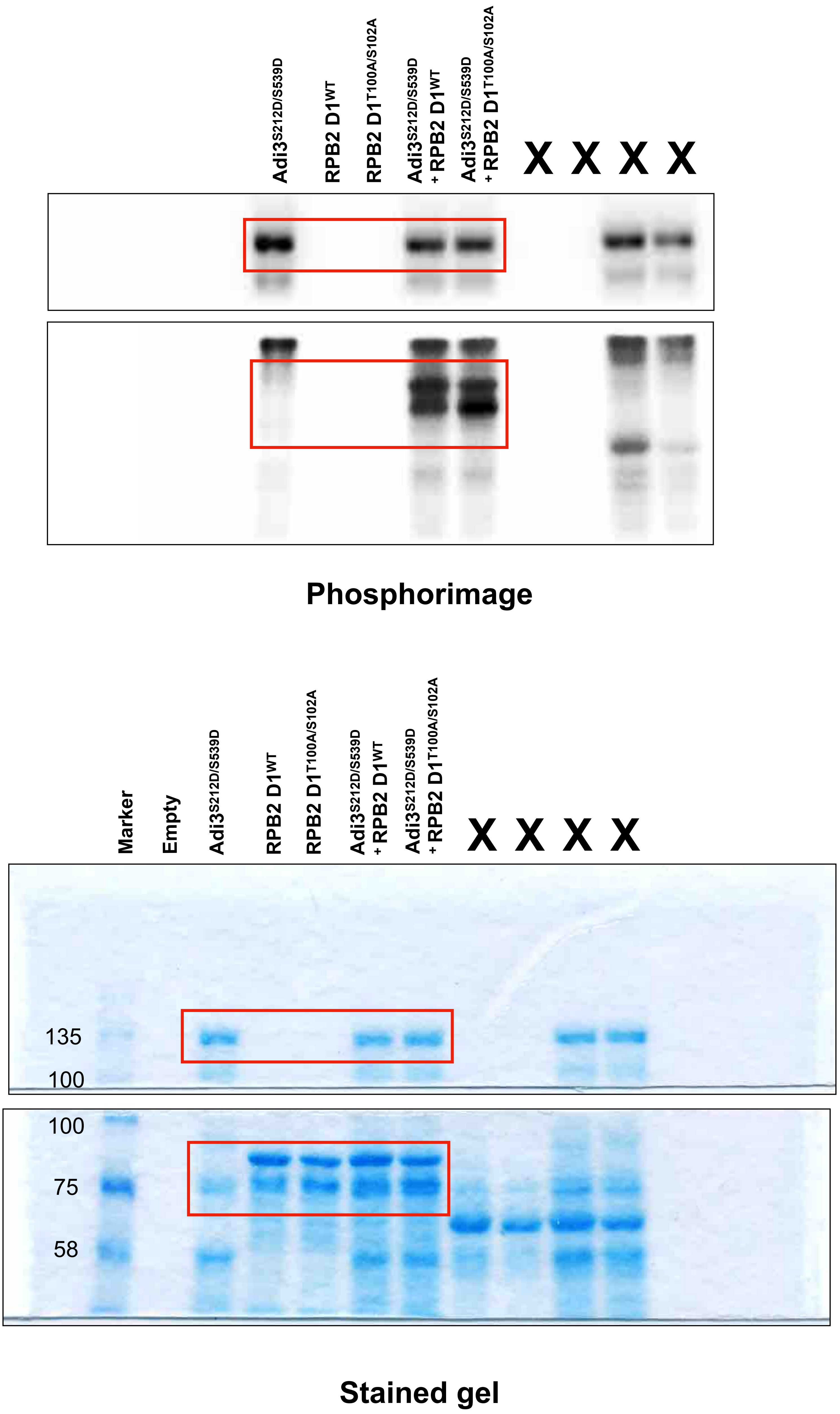
Adi3S212D/S539D
RPB2 D1WT
RPB2 D1T100A/S102A
Adi3S212D/S539D + RPB2 D1WT
Adi3S212D/S539D + RPB2 D1T100A/S102A
X X X X
Phosphorimage
Marker
Empty
Adi3S212D/S539D
RPB2 D1WT
RPB2 D1T100A/S102A
Adi3S212D/S539D + RPB2 D1WT
Adi3S212D/S539D + RPB2 D1T100A/S102A
X X X X
135
100
100
75
58
Stained gel
